# Epigenetic Disruption of the PIWI Pathway in Human Spermatogenic Disorders

**DOI:** 10.1371/journal.pone.0047892

**Published:** 2012-10-24

**Authors:** Holger Heyn, Humberto J. Ferreira, Lluís Bassas, Sandra Bonache, Sergi Sayols, Juan Sandoval, Manel Esteller, Sara Larriba

**Affiliations:** 1 Cancer Epigenetics and Biology Program (PEBC), Bellvitge Biomedical Research Institute (IDIBELL), 08908 L’Hospitalet de Llobregat, Barcelona, Catalonia, Spain; 2 PhD Programme in Experimental Biology and Biomedicine, Center for Neuroscience and Cell Biology, University of Coimbra, Coimbra, Portugal; 3 Laboratory of Seminology and Embryology, Andrology Service-Fundació Puigvert, Barcelona, Catalonia, Spain; 4 Human Molecular Genetics Group, Bellvitge Biomedical Research Institute (IDIBELL), 08908 L’Hospitalet de Llobregat, Barcelona, Catalonia, Spain; 5 Department of Physiological Sciences II, School of Medicine, University of Barcelona, Barcelona, Catalonia, Spain; 6 Institució Catalana de Recerca i Estudis Avançats (ICREA), Barcelona, Catalonia, Spain; Dartmouth Medical School, United States of America

## Abstract

Epigenetic changes are involved in a wide range of common human diseases. Although DNA methylation defects are known to be associated with male infertility in mice, their impact on human deficiency of sperm production has yet to be determined. We have assessed the global genomic DNA methylation profiles in human infertile male patients with spermatogenic disorders by using the Infinium Human Methylation27 BeadChip. Three populations were studied: conserved spermatogenesis, spermatogenic failure due to germ cell maturation defects, and Sertoli cell-only syndrome samples. A disease-associated DNA methylation profile, characterized by targeting members of the PIWI-associated RNA (piRNA) processing machinery, was obtained. Bisulfite genomic sequencing and pyrosequencing in a large cohort (n = 46) of samples validated the altered DNA methylation patterns observed in piRNA-processing genes. In particular, male infertility was associated with the promoter hypermethylation-associated silencing of *PIWIL2* and *TDRD1*. The downstream effects mediated by the epigenetic inactivation of the PIWI pathway genes were a defective production of piRNAs and a hypomethylation of the LINE-1 repetitive sequence in the affected patients. Overall, our data suggest that DNA methylation, at least that affecting *PIWIL2/TDRD1,* has a role in the control of gene expression in spermatogenesis and its imbalance contributes to an unsuccessful germ cell development that might explain a group of male infertility disorders.

## Introduction

Genetic etiologies have only been able to explain about 15% of cases of male infertility [1]. Approximately 4% of men suffer from infertility, with 70% of cases of testicular origin being a consequence of a spermatogenic failure. At least 30% of infertile men suffer from idiopathic infertility of unknown underlying pathophysiology. Mammalian spermatogenesis is a complex and highly regulated developmental process in which mitosis, meiosis and differentiation interact to coordinate the development of a haploid gamete for sexual reproduction. These processes are unique in male germ cell differentiation and depend on precise developmental stage-specific and germ cell type-specific gene expression. Changes in testicular gene expression have been found in spermatogenic failure [2]–[7]. However, the regulatory network that controls germline transcription in mammals is not properly understood. In this context, it has been suggested that DNA methylation may contribute to the control of gene expression programs essential for successful gametogenesis [8].

DNA methylation is an epigenetic process that plays a crucial role in determining the time point and magnitude of gene expression. Unlike the genetic code, the epigenetic code is dynamic and tissue-specific [9]. While the genetic code defines a permanent blueprint of information determining phenotypes and specific traits, the epigenetic code provides a dynamic signalling that is capable of modifying phenotypes according to environmental impacts. Epigenetic regulation is a crucial mechanism for cell fate and survival [10], [11]. In particular, DNA methylation is involved in a wide range of common human diseases [12]–[15]. Within male germ cells, changes in the epigenetic state are critical for silencing transposable elements, imprinting paternal genes, several aspects of meiosis, post-meiotic gene silencing and DNA compaction. Recombinant mouse models identified a profound impact of DNA methylation processing enzymes (DNA methyltransferases, DNMTs) on sperm production. Expression changes of DNMTs in germline stem cells lead to aberrant survival and differentiation [16]. Particularly, a defective DNMT3L results in meiotic failure and impaired spermatogenesis [17]. In addition, DNMT3b mutants reveal a delayed entry into meiosis, resulting in a greatly reduced number of spermatocytes [18].

Abnormal sperm DNA methylation of imprinted genes is associated with spermatogenic impairment [19]–[21], and DNA methylation abnormalities may also involve non-imprinted genes [22]. In this context, it is tempting to speculate that male infertility could be linked to epigenetic alterations, such as abnormal DNA methylation patterns.

It is currently unclear whether DNA methylomes of men with impaired sperm production significantly differ from those presenting a complete and efficient spermatogenic process. To address this matter, we analyzed genome-wide DNA methylation in infertile men with spermatogenic failure. Using the Infinium Human Methylation27 BeadChip technology [23], [24], we obtained an insight into the impact of DNA methylation in secretory male infertility. Among the nearly 600 genes differentially methylated in testis with impaired spermatogenesis compared with tissue with a conserved spermatogenic pattern, we focused on those coding for proteins directly involved in piRNA processing [*PIWIL1* [25]; *PIWIL2* [26]] and associated molecules [*TDRD1* [27], [28], *TDRD9* [29]], due to their potential role in spermatogenic control.

## Materials and Methods

### Subjects of Study

Our study recruited thirty-two infertile patients (aged 30–49 years) due to severe spermatogenic failure (SpF), with a phenotype consistent with non-obstructive (secretory) azoospermia or severe oligozoospermia (<5 million sperm/ml). Only testicular samples with homogeneous phenotypes were selected on the basis of the histological pattern of >20 tubules from the same testicular section; samples with mixed histological patterns were excluded from the study. In addition, five patients with a Sertoli cell-only syndrome (SCO) phenotype were studied as methylation/gene expression controls of somatic cells and nine infertile patients (aged 32–50 years), who were diagnosed with obstructive azoospermia (as a consequence of congenital absence of the vas deferens or a previous vasectomy) and showed conserved spermatogenesis (CS) were studied as methylation/gene expression controls of a complete spermatogenic process (Table 1). Infertile individuals were selected from men referred for couple infertility to the Andrology Service of the Fundació Puigvert. The study was approved by the Institutional Review Board of the Center, and all the participants gave their informed written consent to the procedures of the study.

**Table 1:** Phenotypical and histological description of the testicular samples included in the study.(a).

Patient	Diagnosis	Histology	Semen sperm conc. (million/mL)	Tubular diameter(µm)	Spgonia	Spcyte I	Round Sptid	Elongated Sptid	Sertoli Cells	Johnsen Score
1	OA	CS	0	202.5	25.7	43.6	33.2	26.5	15.3	9.85
2	OA	CS	0	186.7	21.4	28.1	31.3	24.8	9.1	9.70
3	OA	CS	0	200.0	23.6	28.8	30.5	28.3	15.9	9.62
4	OA	CS	0	190.0	20.7	33.7	38.5	33.1	13.5	9.55
5	OA	CS	0	200.0	26.7	28.2	20.3	21.1	13.9	9.30
6	OA	CS	0.02	206.0	25.8	31.8	29.6	21.4	13.1	9.25
7	OA	CS	0.1	229.6	22.8	28.5	16.6	16.0	14.2	9.00
8	OA	CS	0	195.0	20.7	30.5	31.9	12.8	11.5	8.70
9	OA	CS	0	184.0	14.8	29.8	12.5	22.3	8.4	8.65
Mean				199.3	22.5	31.4	27.2	22.9	12.8	
10	SA	SpF (rsMF)	0	200.0	23.4	36.0	19.7	10.1	12.2	8.40
11	SSO	SpF (rsMF)	5	151.7	15.0	27.6	15.5	9.8	11.3	8.40
12	SSO	SpF (rsMF)	0.01	188.0	18.5	33.5	20.4	6.7	19.0	8.30
13	SA	SpF (rsMF)	0.004	191.5	25.1	36.2	19.6	0.7	18.6	7.10
14	SSO + OA	SpF (rsMF)	0	175.0	17.0	24.0	31.0	1.5	6.0	7.00
15	SSO	SpF (rsMF)	3.5	170.0	13.0	20.0	15.0	1.5	11.0	7.00
16	SSO	SpF (rsMF)	5	205.0	20.2	28.6	15.7	0.1	23.3	6.60
17	SSO	SpF (rsMF)	3	148.8	19.7	22.3	15.4	0.3	18.4	6.50
Mean				178.8	19.0	28.5	19.0	3.8	15.0	
18	SA + OA	SpF (scMF)	0	200.6	19.5	22.9	5.6	6.9	15.7	7.10
19	SA	SpF (scMF)	0	168.3	20.4	18.2	5.3	3.1	13.4	7.30
20	SSO	SpF (scMF)	5	181.9	17.1	18.1	10.8	4.1	9.2	7.65
21	SSO	SpF (scMF)	9	216.9	20.8	25.0	4.1	3.0	17.4	6.71
22	SSO	SpF (scMF)	0.8	193.1	13.1	17.2	5.8	2.1	16.7	6.90
23	SA	SpF (scMF)	0	170.0	19.5	17.6	4.0	2.1	14.1	6.70
24	SA	SpF (scMF)	0.005	156.9	17.9	21.6	4.2	1.2	8.6	5.80
25	SSO	SpF (scMF)	0.4	196.8	24.8	21.1	1.9	0.5	11.8	5.20
26	SA	SpF (scMF)	0	190.0	23.0	30.0	0.0	0.0	11.0	5.00
27	SA	SpF (scMF)	0	190.0	17.6	18.2	0.0	0.0	20.0	5.00
28	SA	SpF (scMF)	0	190.0	22.5	39.6	0.0	0.0	16.0	5.00
29	SA	SpF (scMF)	0	168.3	15.1	12.5	2.3	1.7	12.2	6.90
30	SA	SpF (scMF)	0	175.0	15.9	10.8	0.0	0.0	10.1	4.73
31	SSO	SpF (scMF)	0.5	191.0	18.8	6.6	2.0	1.3	12.8	5.75
32	SSO	SpF (scMF)	0.015	190.0	23.0	6.0	5.0	5.0	11.0	4.40
Mean				185.3	19.3	19.0	3.4	2.1	13.3	
33	SA	SpF (sgMF )	0.009	166.7	12.7	23.1	18.7	10.6	15.3	8.10
34	SSO	SpF (sgMF )	0.02	148.3	10.8	17.8	7.4	10.2	7.4	6.80
35	SSO	SpF (sgMF )	0.1	164.2	12.0	14.0	9.1	8.0	7.6	6.75
36	SA	SpF (sgMF )	0	171.9	13.1	15.7	10.7	10.9	16.0	5.40
37	SSO	SpF (sgMF )	0.6	136.3	6.0	6.7	1.7	0.2	6.5	4.50
38	SA	SpF (sgMF )	0	153.1	8.3	12.6	0.0	0.0	6.6	3.45
39	SA	SpF (sgMF )	0	97.5	8.5	0.2	0.0	0.0	5.2	2.80
40	SA	SpF (sgMF )	0	115.0	2.8	2.3	0.2	0.0	7.7	3.80
41	SSO	SpF (sgMF )	0.8	156.2	1.1	1.2	1.8	1.8	28.1	2.40
Mean				145.5	8.3	10.4	5.5	4.6	11.1	
42	SA	SCO	0	163.3	0.0	0.0	0.0	0.0	28.5	2.00
43	SA + OA	SCO	0	170.0	0.0	0.0	0.0	0.0	17.0	2.00
44	SA	SCO	0	165.0	0.0	0.0	0.0	0.0	19.5	2.00
45	SA	SCO	0	165.0	0.0	0.0	0.0	0.0	20.7	2.00
46	SA	SCO	0	165.0	0.0	0.0	0.0	0.0	19.1	2.00
Mean				165.6	0.0	0.0	0.0	0.0	21.0	

Abbreviations: conc., concentration; CS, conserved spermatogenesis; OA, obstructive azoospermia; SA, secretory azoospermia; SCO, Sertoli.

cell only syndrome; Spcyte, spermatocyte; SpF, spermatogenic failure; Spgonia, spermatogonia; Sptid, spermatid; SSO, severe secretory.

oligozoospermia; sgMF, maturation failure at spermatogonia level; scMF, maturation failure at spermatocyte level; rsMF, maturation failure at round.

spermatid level.

(a) The mean number of the different type of cells per tubule is given in each group.

The clinical procedures for infertile patients included medical history, physical examination, semen analyses (performed in accordance with World Health Organization guidelines [30]) and hormonal study. Concentrations of FSH generally reflected the findings of the testicular histology, although some patients showing primary spermatocyte arrest or hypospermatogenesis had normal FSH (data not shown). Spermiograms included volume, pH, sperm concentration, motility, vitality, morphology and fructose and citrate levels in seminal plasma. The testicular biopsy was obtained when necessary to confirm the clinical diagnosis and for sperm retrieval (TESE) and cryopreservation purposes.

The routine genetic study for all non-obstructive samples included karyotype and analysis of chromosome Y microdeletions, the latter performed according to the European guidelines [31], [32]. Men with a chromosomal aberration or a Y-chromosome microdeletion were not included in the study.

### Testicular Samples

Testicular biopsies of infertile men were obtained under local anesthesia through a small incision. Each specimen was divided into three aliquots, one piece (≈10–20 mg) was fixed in Bouin’s solution and reserved for histological analysis, a second aliquot (≈100–200 mg) processed for sperm extraction, and the third (≈10 mg) was immediately transferred to liquid nitrogen and then stored at –80°C until used for molecular analysis.

### Histological Analysis

Fixed testicular biopsies were cut into 5-µm sections and stained with hematoxylin–eosin. Germ cells of the different levels of maturation (spermatogoniae, spermatocytes I, round spermatids and elongated spermatids) and Sertoli cells were quantified, and the average number per tubule was calculated after analysis of at least 15–20 cross-sectioned tubules/testis. Assessment of the spermatogenic status and the severity of the alteration is shown by a modified Johnsen score (JS) [33], calculated on the basis of the number of different cell types per tubule.

Using this strategy we confirmed the diagnosis of SCO and CS phenotypes. With respect to SpF patients, eight of them presented maturation failure at the round spermatid level (rsMF), fifteen at the spermatocyte level (scMF) and nine at the spermatogonia level (sgMF), due to the presence of a diminished number of this specific stage and the subsequent germ cell stages in their tubules compared with CS samples (Table 1).

### Spermatozoa Isolation and DNA Extraction

Semen samples obtained from normozoospermic men were collected and allowed to liquefy for 30 min. Before the standard swim-up separation technique, whole semen was centrifuged on a 25% Percoll gradient (20 minutes) to discard somatic cell contamination, further ensuring the purity of the sperm population. The swim-up procedure results in selection of spermatozoa with good motility.

Sperm DNA was extracted with an user-developed version of the QIAamp® DNeasy&Tissue Kit purification protocol (Qiagen). Fresh washed (in PBS) sperm was incubated 1∶1 with a lysis buffer containing 20 mM TrisCl (pH 8), 20 mM EDTA, 200 mM NaCl and 4% SDS, supplemented prior to use with 100 mM DTT and 250 ug/ml Proteinase K. Incubation was performed for 4 hours at 55°C with frequent vortexing. Prior to processing in the columns, 200 ul of absolute ethanol and 200 ul of the kit-provided lysis buffer were added to the samples. Then, purification was performed according to kit instructions.

### DNA Methylation-specific Array

Genomic DNA was extracted from testicular biopsies by using the Wizard Genomic DNA Purification kit (Promega, Madison, USA). DNA methylation profile was assessed using the Infinium Human Methylation27 BeadChip (Illumina, San Diego, USA), which assays DNA methylation levels at 27,578 CpG sites. Briefly, DNA was quantified by Quant-iT™ PicoGreen dsDNA Reagent (Invitrogen, Carlsbad, USA) and the integrity was analyzed in a 1.3% agarose gel. Bisulfite conversion of 600 ng of each sample, which results in unmethylated cytosines being converted to uracils, whereas methylated cytosines are not converted, was performed according to the manufacturer’s recommendation for the Illumina Infinium Assay. Effective bisulfite conversion was checked for three controls that were converted simultaneously with the samples. The intensities of the images were extracted and normalized using GenomeStudio (V2010.3, Illumina) software. The methylation score of each CpG was represented as a beta (β) value. The threshold for concluding differential methylation of probes was set at an average delta β value >0.1.

### Bisulfite Sequencing

Genomic DNA was bisulfite-modified using the EZ DNA Methylation-Gold Kit (Zymo Research, Orange, USA) according to the manufacturer’s protocol. The methylation status of selected regions was analyzed by bisulfite genomic sequencing. Bisulfite-converted DNA was amplified (Table S1) and subsequently cloned using the pGEM-t easy kit (Promega, Madison, USA). At least eight independent clones were analyzed in an automated ABI Prism 3700 sequencer (Applied Biosystems, Carlsbad, USA).

### Pyrosequencing

Amplification primers (Table S1) and sequencing settings were designed using a PyroMark assay design (V2.0.01.15; Qiagen). LINE-1 was quantified using the PyroMark Q96 LINE-1 assay (Qiagen). PCR was performed with primers biotinylated to convert the PCR product to single-stranded DNA templates. The Vacuum Prep Tool (Biotage, Uppsala, Sweden) was used to prepare single-stranded PCR products according to the manufacturer’s instructions. Pyrosequencing reactions and methylation quantification were performed using the PyroMark Q96 System (Qiagen).

### Gene Expression Quantification

Total RNA was obtained from the testicular biopsy using the Absolutely RNA Miniprep Kit (Stratagene, La Jolla, CA), according to the instructions provided by the manufacturer. Furthermore, small RNA-containing total RNA was additionally obtained with a mirVana miRNA Isolation Kit (Ambion) from an extra portion of the testicular biopsy whenever this was possible. The quality of RNA was assessed using the Agilent 2100 Bioanalyzer (Agilent Technologies, Waldbronn, Germany). Testicular RNA samples included in the study had a 28S/18S ratio >1.3 and an RIN value >7.5. Single-stranded cDNA was obtained by reverse transcription (RT) of 500 ng of RNA using random hexamer primers and the High Capacity cDNA Reverse Transcription Kit (Applied Biosystems).

Quantitative real-time PCR (qPCR) reactions were performed on an ABI 7300 real-time PCR system (Applied Biosystems) using gene-specific TaqMan Assays (*PIWIL2*: Hs00216263_m1; *TDRD1*: Hs00229805_m1; *PGM1*: Hs00160062_m1) and custom-designed small RNA TaqMan Assays (Applied Biosystems). Negative controls without template were included in each set of PCR assays as well as a calibrator sample to compare the change in expression of a nucleic acid sequence against the expression in all samples in the same study. *PGM1* was previously selected as an appropriate reference gene among ten candidate genes tested (data not shown) for *PIWIL2* and *TDRD1* data normalization in our study, showing similar Ct values to the ones obtained from target genes, no statistical differences in expression among groups_(Kruskal-Wallis test) and low M-value (GeNorm software; [34]) indicating stable expression among samples. For piRNA expression analysis the arithmetic mean value of Ct values of *RNU48*, *RNU19* and *RNU6B* was used for normalization.

Patient and control group samples were always analyzed as paired samples in the same analytical run in order to exclude between-run variations. Real-time qPCR data were pre-processed using the 2^−ΔΔCt^ strategy and stored in SDS 2.1 software (Applied Biosystems). Expression levels are shown as relative quantification (RQ) values.

### Statistical Analysis

Statistical analyses were performed using SPSS 12.0 software (SPSS Inc, Chicago, Illinois). The nonparametric Mann-Whitney U test was used to analyze differences in absolute expression and methylation level in SpF patient groups compared with controls. Pearson product-moment correlation coefficients were calculated to determine the correlation between the methylation status, expression ratios of the target genes and the various histological parameters in patient groups and controls. A value of p<0.05 was considered significant. Gene Ontology (GO), pathways enrichment analysis was performed using the Database for Annotation, Visualization and Integrated Discovery (DAVID; v6.7).

### piRNA Target Identification

The complete set of piRNA sequences was obtained from piRNA bank (http://pirnabank.ibab.ac.in) and aligned (BLAT) to the reference genome. Subsequently, the promoter regions (transcription start site +/−2 kb) of the 580 differentially methylated genes were analyzed for the presence of piRNA complementarity. Promoters with sequence identity of 100% to any piRNA in the data set were regarded as potential regulative target.

## Results

### DNA Methylation Profiles Distinguish Male Infertility Disorders from Physiological Germ Cell Development

In order to identify the genome-wide DNA methylation changes associated with severe germ cell development deficiencies in the testis we used a DNA methylation bead-assay covering 27,578 CpG sites in the genome [23], [24], [35]. As the probes are almost exclusively located in promoter regions, the array gives a comprehensive overview of 14,495 individual genes. The reproducibility and sensitivity of the array has been described elsewhere [23], [24], [35]. Using this platform, we analyzed the methylation profile of testis with conserved spermatogenesis (CS) (n = 2), spermatogenic failure (SpF) samples (two of them -sample no. 25 and 28- presented maturation failure at the spermatocyte level [scMF] and two -sample nos. 10 and 13- at the round spermatid level [rsMF]) and Sertoli cell-only syndrome patients (SCO) (n = 2), the latter completely lacking germ cells in the testicular tubules. CS and SpF samples show similar numbers of spermatogoniae and spermatocytes in the tubule (Table 1).

**Figure 1:**
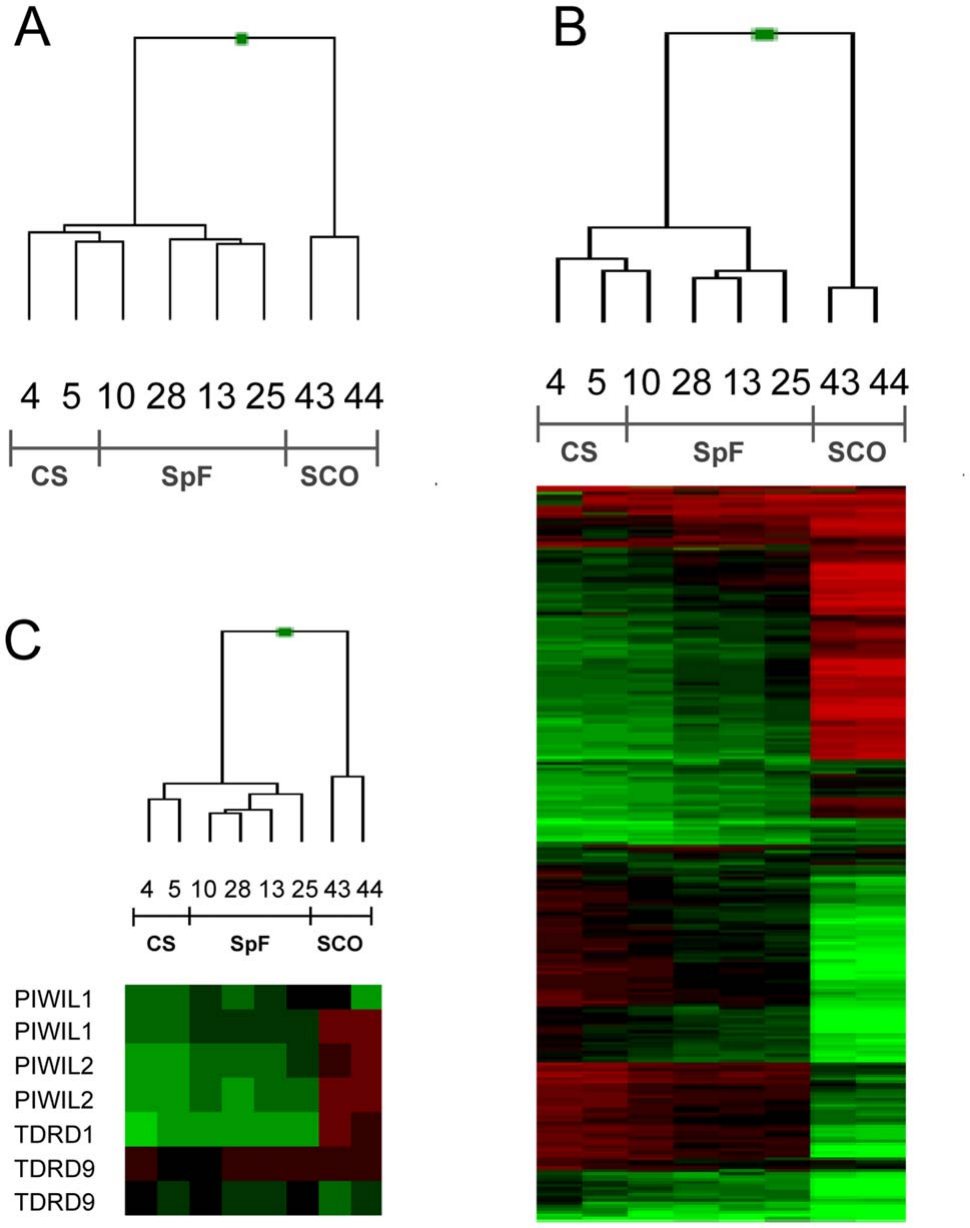
DNA methylation microarray analysis determined disease-associated profiles. (A) Unsupervised hierarchical clustering separated testis with a complete absence of germ cells (SCO) from those with the presence of germ cell lineage, and testis with conserved spermatogenesis (CS) from those with spermatogenic failure (SpF) human samples. (B) Hierarchical clustering of CS, SpF and SCO samples, displaying the 633 CpG sites differentially methylated between CS and SpF samples. (C) Hierarchical clustering of *PIWIL1/2* and *TDRD1/9* involved in the piRNA processing machinery. Sample number corresponding to that in Table 1 is also indicated.

Comparing all CG sites interrogated by the array platform, SpF samples had a highly similar profile to the CScontrol (r^2^ = 0.99). However, unsupervised hierarchical clustering revealed a distinct methylation profile of the three SpF patient samples that entirely lacked elongated spermatids, unlike the CS controls (samples no. 13, 25 and 28; Table 1). Furthermore, the methylation profile of the SpF sample no. 10 was clustered with the CS control samples (Fig. 1A). The SCO specimens had highly variable methylation levels compared with the other groups, reflecting the somatic origin of the Sertoli cells and the specific methylation patterns of germline and somatic tissues. We were also able to identify 633 differentially methylated sites (DMSs; average delta β value >0.1) (327 hypomethylated and 306 hypermethylated) in the three clustered SpF samples relative to the CS control tissue, representing 580 different genes (comprises 4% of the tested genes) (Fig. 1B, Table S2). Interestingly, while most of the probes present on the array were located in CpG islands (73%), the DMSs identified were significantly enriched in CpG-poor promoters (64%; Chi-square test, p<0.01). From a biological ontology point of view, hypermethylated genes are enriched in functions directly related to germline processes, such as germline stem-cell maintenance (Fisher’s exact test, p = 1.6×10^−4^), reproductive cellular process (Fisher’s exact test, p = 0.018) and gamete generation (Fisher’s exact test, p = 0.03) (Table S3). In particular, hypermethylation of *PIWIL1*, *PIWIL2*, *SPATA16*, *MSH4*, *INSL3*, *CNGA1*, *FANCG* and *HIST1H1T* contributed to the enrichment in the biological process of male gamete generation. Furthermore, other germline-specific genes such as *PAGE1* and *XAGE3/5* were found to be differentially methylated in SpF patient samples relative to CS (Table S2).

It is of particular note that genes involved in the biogenesis of PIWI-associated RNAs (piRNAs), such as the differentially methylated in the array *PIWIL1/2* and their associated molecules *TDRD1/9*, were able to cluster normal tissue and patient samples (including sample no.10) separately, suggesting that hypermethylation of these genes is a disease-associated event (Fig. 1C).

### Promoter Hypermethylation-associated Transcriptional Silencing of *PIWIL2* and *TDRD1* in Infertile Males with Spermatogenic Failure

**Figure 2:**
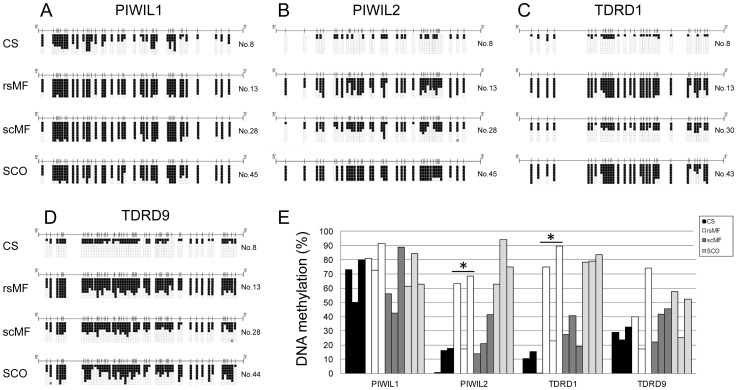
*PIWIL2* and *TDRD1* become more methylated in human infertility syndromes. Bisulfite sequencing of the piRNA processing genes *PIWIL1* (A), *PIWIL2* (B), *TDRD1* (C) and *TDRD9* (D). Black and white squares indicate CpG methylation and unmethylated sites, respectively. One representative sample of a large difference between testis with conserved spermatogenesis (CS), maturation failure at the spermatocyte (scMF) or at the round spermatid (rsMF) stage, and in Sertoli cell-only syndrome (SCO) are displayed. Sample number corresponding to that in Table 1 is also indicated. (E) Methylation level of gene promoters of *PIWIL1, PIWIL2*, *TDRD1* and *TDRD9* in testis with conserved spermatogenesis (CS), maturation failure at the spermatocyte (scMF) or at the round spermatid (rsMF) stage, and in Sertoli cell-only syndrome (SCO) samples. Independent data are also shown in Figure S1 and S2. Significant differences compared to CS samples are indicated (*).

The disruption of genes associated with the piRNA processing machinery has been directly related to spermatogenic failure due to maturation arrest, resulting in male sterility in mouse models. As genome-wide analysis of patient samples with spermatogenic failure identified deregulation of genes involved in piRNA production, we aimed to validate these genes in a larger cohort of samples. To assess the impact of DNA methylation on genes involved in the piRNA processing machinery, we performed bisulfite genomic sequencing of the promoter regions of *PIWIL1/2* and *TDRD1/9* in three CS samples, six SpF samples (three rsMF and three scMF) and three SCO samples (Fig. 2 and Fig. S1 and S2). Differences in DNA methylation between SpF and CS samples were observed in all the genes analyzed, the magnitude being most striking for *PIWIL2* and *TDRD1*. Both genes displayed minimal or no promoter methylation in CS normal tissue and a great increase in rsMF (Student’s t test <0.05) samples. *TDRD9* also exhibited an elevated level of methylation, although we had already detected increased levels in normal tissue. This was even more evident for *PIWIL1*, for which half of the CpG sites analyzed were found to be methylated in CS samples. As expected, we observed striking differences between CS testis tissue and SCO samples, which is consistent with the germline-specific expression associated with the analyzed genes. Here, hypermethylation was detected in all five genes, probably due to the total absence of germ cells, in SCO specimens.

**Figure 3:**
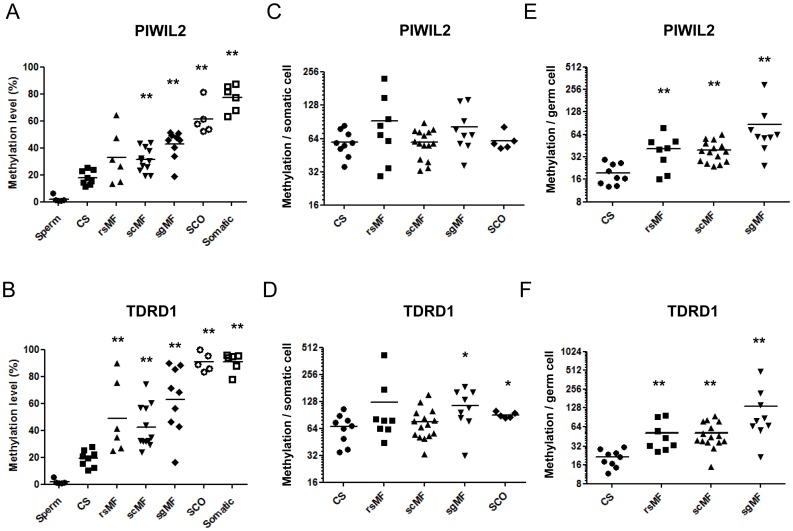
SpF samples show a *PIWIL2* and *TDRD1* hypermethylation pattern. Methylation levels of *PIWIL2* (A) and *TDRD1* (B) in mature spermatozoa (sperm), testis with a conserved spermatogenic pattern (CS), maturation failure at the round spermatid (rsMF), the spermatocyte (scMF) and the spermatogonia (sgMF) stages, Sertoli cell-only syndrome (SCO) and somatic tissue measured by pyrosequencing. The black bar indicates the mean methylation level. Methylation per cell profiling of *PIWIL2* and *TDRD1*, displayed as methylation per somatic cell (x100) (C, D) and methylation per germ cell (x100) (E, F) in testis with conserved spermatogenesis (CS), maturation failure at the round spermatid (rsMF), the spermatocyte (scMF) and the spermatogonia (sgMF) stages and Sertoli cell-only syndrome (SCO). The horizontal bar displays the mean cellular expression level. Significant differences from the control are indicated: *p<0.05; **p<0.01.

To confirm the hypermethylation of *PIWIL2* and *TDRD1* in SpF*-*affected patients, locus-specific pyrosequencing was performed in a larger validation patient cohort. In detail, nine normal CS control, thirty-two SpF (rsMF, scMF, sgMF) affected patient samples and five SCO specimens were analyzed to detect the CpG site immediately upstream of the transcription start site. To gain a better insight into the tissue specificity of *PIWIL2* and *TDRD1*, we also included five samples from mature swim up-selected spermatozoa and six somatic tissues from colon, breast, blood, skin, lung and brain in the study. As expected, spermatozoa exhibited an absence of DNA methylation in both genes, whereas both promoters were heavily methylated in all somatic tissues (Fig. 3A and B). In SpF samples, we were able to validate a significant increase in promoter methylation (Mann-Whitney test, p<0.01) of *PIWIL2* and *TDRD1* (Fig. 3A and B). It is of note that SCO samples and somatic tissues had similar *TDRD1* DNA methylation levels, whereas *PIWIL2* methylation is reduced in SCO when compared to somatic tissues (Mann-Whitney test, p = 0.015; Fig. 3A and 3B). Furthermore, to have a better insight into methylation level related to the testicular sample cell composition, *PIWIL2* and *TDRD1* methylation data was first divided by the proportion of somatic cells per tubule (Fig. 3C and D). The number of somatic cells was inferred from the number of Sertoli cells quantified per tubule in each testicular sample, as the Sertoli cells represent the 30% of somatic cells related to one testicular tubule. As no SpF sample was observed to present hyperplasia of Leydig cells it is assumed that the somatic cell number was constant among CS and SpF samples. We observed no statistical difference in methylation per somatic cell among the different subgroups of the study. Additionally *PIWIL2* and *TDRD1* methylation data was divided by the proportion of germ cells per tubule obtaining a hypermethylation profile, being more considerable as the maturation failure affects an earlier germline stage (CS<rsMF<scMF<sgMF; Mann-Whitney test p<0.001; Fig. 3E and F). Altogether this analysis suggest that the changes in the tissular methylation pattern observed in SpF samples could not be exclusively explained by the proportion of somatic cells in the testicular sample but it is the result of the sum of the increased proportion of somatic cells and, also, of the increase of individual methylation of germ cells.

**Figure 4:**
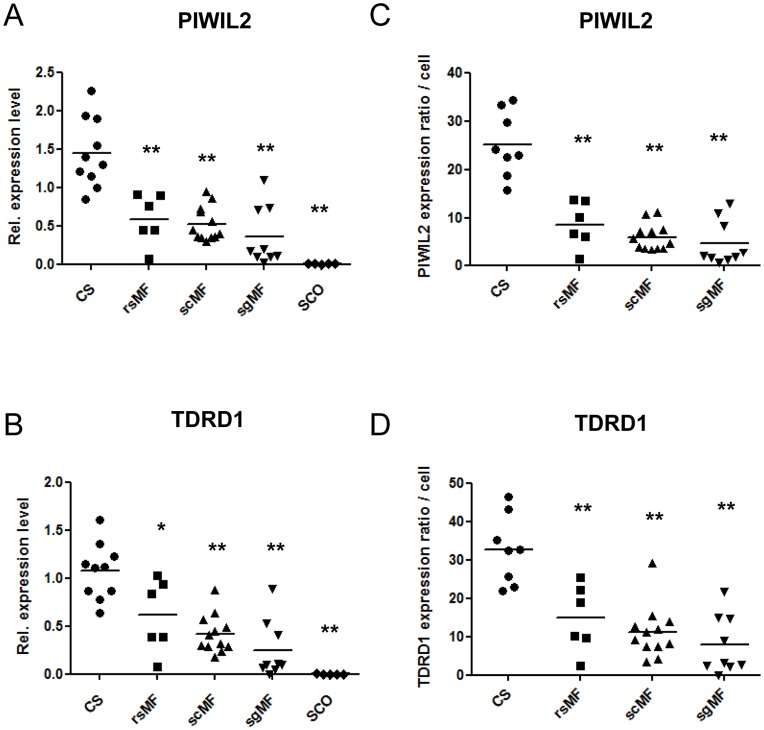
*PIWIL2* and *TDRD1* hypermethylation is negatively associated with *PIWIL2* and *TDRD1* transcript levels in SpF samples. Tissular expression profiling of *PIWIL2* (A) and *TDRD1* (B) by quantitative real-time qPCR in testis with conserved spermatogenesis (CS), maturation failure at the round spermatid (rsMF), the spermatocyte (scMF) and the spermatogonia (sgMF) stages and Sertoli cell-only syndrome (SCO). Expression levels relative to *PGM1* are shown. Expression per cell profiling of *PIWIL2*, displayed as expression ratio per spermatogonia/spermatocyte (x1000) (C) and expression per cell of *TDRD1*, displayed as expression ratio per spermatocyte (X1000) (D) in testis with conserved spermatogenesis (CS), maturation failure at the spermatocyte (scMF), the round spermatid (rsMF), and the spermatogonia (sgMF) stages. The horizontal bar displays the mean cellular expression level. Significant differences from the control are indicated: *p<0.05; **p<0.01.

Most importantly, the hypermethylation observed in patients was accompanied by reduced transcript levels (Fig. 4A and B). Both genes had significantly less transcript in the SpF patient samples than in the CS control tissue (Mann-Whitney test, p<0.01). Strikingly, tissular gene methylation and expression level were highly significantly and negatively correlated for *PIWIL2* (Pearson’s correlation, r = −0.74; p<0.0001) and *TDRD1* (Pearson’s correlation, r = −0.76; p<0.0001). We additionally analyzed the germ cell–specific transcript levels per cell in SpF subgroups compared to CS controls in order to exclude the differences in gene expression due to changes in testicular cellularity and to determine whether transcript level per cell is also altered in SpF. Values of transcript amount per cell, in arbitrary units, were obtained for each testicular sample by dividing the *PIWIL2/TDRD1* expression values by the proportion of expressing germ cell stages present in a seminiferous tubule of the sample, being spermatogoniae and primary spermatocytes the germ cell stages that predominantly express *PIWIL2,* whereas spermatocytes predominantly express *TDRD1* in the testis (GermSAGE; http://germsage.nichd.nih.gov) (Fig. 4C and D). Significant differences in cellular transcript levels were found for both genes between SpF patients and controls (Mann-Whitney test, p<0.01). Interestingly, the expression level per cell and the gene methylation were also highly significantly and negatively correlated for *PIWIL2* (Pearson’s correlation, r = −0.70; p<0.0001) and *TDRD1* (Pearson’s correlation, r = −0.62; p<0.0001).

In order to assess whether there was an association between molecular and histological data and to confirm any physiological relevance, using all the samples in the study, we calculated the correlations between the normalized gene expression values, pyrosequencing data and histological parameters such as the number of each type of cell from the germline, Sertoli cell number and JS count (Table 2). All the histological parameters, with the exception of the Sertoli cell number, were positively correlated with *PIWIL2* and *TDRD1* expression values, being remarkable the correlation coefficient between the cellular transcript levels of *PIWIL2* and the number of elongated spermatids in the tubule (r = 0.82; p<0.0001), whilst a negative correlation coefficient was obtained with their DNA methylation levels.

**Table 2:** Pearson correlation coefficients and adjusted p values (r;p) between the molecular and the histological parameters for all the samples analysed.

	Spermatogonia	Spermatocyte I	Roundspermatid	Elongatedspermatid	Sertolicells	germcells/tubule	JS
Pyroseq *PIWIL2*	−0.619 *p*<**0.0001**	−0.647 *p*<**0.0001**	−0.465 *p* = **0.001**	−0.533 *p<* **0.0001**	0.273*p* = 0.067	−0.656 *p*<**0.0001**	−0.705 *p*<**0.0001**
Pyroseq *TDRD1*	−0.696 *p*<**0.0001**	−0.718 *p*<**0.0001**	−0.414 *p* = **0.004**	−0.492 *p = * **0.001**	0.228*p* = 0.128	−0.671 *p*<**0.0001**	−0.687 *p*<**0.0001**
RQ *PIWIL2*	0.613 *p*<**0.0001**	0.683 *p*<**0.0001**	0.541 *p*<**0.0001**	0.674 *p*<**0.0001**	−0.266 *p* = 0.097	0.724 *p*<**0.0001**	0.711 *p*<**0.0001**
RQ *TDRD1*	0.666 *p*<**0.0001**	0.766 *p*<**0.0001**	0.597 *p*<**0.0001**	0.635 *p*<**0.0001**	−0.208 *p* = 0.198	0.774 *p*<**0.0001**	0.734 *p*<**0.0001**
RQ *PIWIL2*/cell	0.566 *p*<**0.0001**	0.667 *p*<**0.0001**	0.677 *p*<**0.0001**	0.819 *p*<**0.0001**	−0.216 *p* = 0.181	0.788 *p*<**0.0001**	0.764 *p*<**0.0001**
RQ *TDRD1*/cell	0.641 *p*<**0.0001**	0.595 *p*<**0.0001**	0.649 *p*<**0.0001**	0.732 *p*<**0.0001**	−0.273 *p* = 0.088	0.749 *p*<**0.0001**	0.700 *p*<**0.0001**

Significant differences are indicated in bold.

### Defects in the Establishment of piRNA-directed DNA Methylation Patterns in Impaired Sperm Production

**Figure 5:**
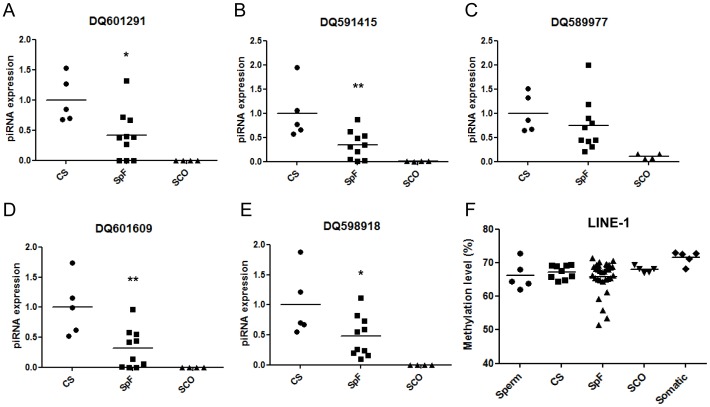
Downregulation of *PIWIL2* and *TDRD1* is associated with piRNA reduction and LINE-1 hypomethylation. (A–E) Expression levels of selected piRNAs in testis with conserved spermatogenesis (CS), spermatogenic failure (SpF), Sertoli cell-only syndrome (SCO) phenotypes, measured by qPCR. Expression levels relative to *RNU48*, *RNU19* and *RNU6B* are shown. (F) Methylation profiling of LINE-1 in mature spermatozoa (sperm), testis with conserved spermatogenesis (CS), spermatogenic failure (SpF), Sertoli cell-only syndrome (SCO) phenotypes and somatic tissue, measured by pyrosequencing. Significant differences from the control are indicated: *p<0.05; **p<0.01. Mean expression levels are depicted by horizontal bars.

We examined the potential consequences of *PIWIL2* and *TDRD1* repression in patient samples with spermatogenic defects. First, we determined the expression of five primary piRNAs with strictly restricted germline expression [36] (piRNABank accession no. DQ601291, DQ591415, DQ601609, DQ589977, DQ598918) in five normal CS control, ten SpF (rsMF n = 3, scMF n = 7) affected SpF samples and four SCO specimens. Consistently with the somatic origin, minimal or absent expression of the analyzed piRNAs was observed in SCO samples (Fig. 5A–E). All piRNAs were downregulated in SpF samples relative to CS and were directly and inversely correlated with *PIWIL2*/*TDRD1* expression (range of Pearson’s correlation, r = 0.652–0.782; p<0.01) and DNA methylation (range of Pearson’s correlation, r = −0.595–−0.767; p<0.01), respectively, as well as with the severity of spermatogenic impairment measured as the JS value (range of Pearson’s correlation, r = 0.643–0.756; p<0.001). Remarkably, expression of DQ601291, DQ591415 and DQ601609 was abolished in those samples with complete meiotic arrest (samples no. 26 and 27) suggesting that they were postmeiotic piRNAs and that the absence of expression might be at least partially due to the loss of postmeiotic germ cells. However, this was not the case for DQ589977 and DQ598918: they had a moderate level of expression in samples with meiotic arrest, suggesting a premeiotic expression of these piRNAs. Thus, we additionally analyzed the germ cell–specific levels of both piRNAs (DQ589977 and DQ598918) per cell in SpF samples compared to CS controls in order to obviate the differences in piRNA expression due to changes in tissular germ cell composition. Transcript levels per cell, in arbitrary units, were obtained for each testicular sample by dividing the piRNA expression value by the proportion of spermatogoniae and primary spermatocytes present in a seminiferous tubule of the sample. Interestingly, piRNA expression per cell positively correlated with *PIWIL2*/*TDRD1* expression (range of Pearson’s correlation, r = 0.660–0.748; p<0.01), *PIWIL2*/*TDRD1* expression per cell (range of Pearson’s correlation, r = 0.663–0.711; p<0.01) and the severity of spermatogenic impairment measured as the JS value (range of Pearson’s correlation, r = 0.602–0.714; p<0.001). Negative correlation was observed between piRNA expression per cell and DNA methylation (range of Pearson’s correlation, r = −0.553–−0.696; p<0.01).

PIWI/piRNAs complexes were shown to methylate DNA to silence transposons in male germline stem cells. Thus, as the piRNA-related machinery is directly involved in the regulation of repetitive elements, we profiled the methylation status of LINE-1. In accordance with the repression of *PIWIL2* and *TDRD1*, we observed hypomethylation of LINE-1 sequences using pyrosequencing technology in five out of the thirty-two patients suffering spermatogenetic failure (Fig. 5F), three of whom were scMF and two sgMF (sample nos. 21, 23, 24, 35 and 39).

In addition to repetitive elements, piRNAs also showed the ability to guide DNA methylation and thereby regulate the expression of protein-coding and non-coding genes. In order to identify the DNA methylation changes of potential piRNA target genes associated with spermatogenic failure, we screened the promoter regions (transcription start site +/−2 kb) of the 580 genes differentially methylated in SpF for sequence overlap with piRNAs. We identified seven genes that were differentially methylated between normal and patient samples and also found complementarity to piRNAs sequences within their promoter (Table S4), which implies that they might be directly targeted by piRNA-mediated regulation. In detail, interleukin 16 (*IL16*), kallikrein 1 (*KLK1*), G protein-coupled receptor 156 (*GPR156*), histone cluster 1 H2aa (*HIST1H2AA*), RAB24, member RAS oncogene family (*RAB24*), sphingomyelin phosphodiesterase 3 (*SMPD3*) and dolichyl-phosphate mannosyltransferase polypeptide 1 (*DPM1*) showed piRNA sequence identity in their promoter region, suggesting an association of piRNA deregulation with the promoter methylation and gene expression.

## Discussion

Although, several studies have reported the impact of an aberrant DNA methylation [16]–[18], [37] on spermatogenesis and fertility in mouse model systems, so far none of this knowledge has been transferred to impaired sperm production in humans. Consequently, we investigated the methylation profiles, especially of piRNA-associated proteins, and their potential consequences in defective human spermatogenesis.

We screened for global DNA methylation changes in diseased specimens using an array technology capable of profiling the DNA methylation level of 14,495 genes. It has been described that although the vast majority of methylation acquisition in male germ cells is completed in primordial germ cells, before birth, changes of DNA methylation continue to occur at a reduced number of CpGs during spermatogenesis before pachytene [38]. In order to distinguish SpF-associated differences in DNA methylation from physiological spermatogenic process, we selected samples that had similar number of cells from the earliest stages of the germline. With this strategy, not only did we detect more than 600 differentially methylated CpG sites, which allowed us to separate CS and SpF samples, but also we identified that the affected promoters were enriched in genes involved in germline function and spermatogenesis, suggesting that gene repression by hypermethylation of germline specific genes is probably a driver of infertility. Detecting similar numbers of gains and losses within differentially methylated positions rules out the possibility of unidirectional methylation changes, the converse of the global hypomethylation observed in other germ cell-related pathological diseases such as seminoma [39]. Interestingly, in agreement with our results, some of the SpF-hypermethylated genes were previously found hypermethylated in DNA from semen with poor sperm concentration (i.e. *SFN* gene and the maternally imprinted genes *PLAGL1* and *DIRAS3*) [22]. The SCO specimens exhibited a different profile of methylation compared with control and SpF samples. This could reflect the distinct sample compositions, whereby SCO samples almost exclusively contained somatic cells, whereas SpF samples showed impaired spermatogenesis and germ cells still present. However, we need to bear in mind that additional phenotypic changes related to somatic cells are observed in SCO, such as a greater number of Sertoli cells (Table 1). Thus, an aberrant pattern of methylation associated with this extremely severe phenotype of secretory infertility could not be ruled out.

Among the genes that are hypermethylated in the SpF phenotype, genes that encode PIWI family members and their associated proteins involved in piRNA processing such as *PIWIL1/2*, *TDRD1/9* were able to cluster normal tissue and patient samples separately. These results and the fact that impaired expression of these genes leads to sterility in animal mouse models [26]–[28] encouraged us to analyze this subgroup of genes in more detail. We identified and confirmed that *PIWIL2* and *TDRD1* were hypermethylated in SpF specimens. The increase in methylation in SpF patients could be partially explained by the increase proportion of somatic cells in the samples but interestingly, an additional statistically significant increase in the methylation level of germ cells was observed (Fig. 3 and F) in SpF subphenotypes when compared to CS samples, being more pronounced in the sgMF subphenotype. The gain in methylation was shown to be significantly correlated with lower *PIWIL2* and *TDRD1* expression level analyzing the entire tissue and more importantly the expression per cell. The remarkable correlation coefficient between the *PIWIL2* transcript levels per cell and the number of elongated spermatids in the testicular tubule additionally underlines the determinant role of *PIWIL2* expression in the progression of the spermatogenic process. Moreover, its potential use as a surrogate marker for the presence of full spermatogenesis in severe non-obstructive infertile patients should be additionally considered.

The abnormal methylation of *PIWIL2,* but not of *PIWIL1,* in spermatogenic impairment suggests that proper methylation is essential in the early stages of spermatogenesis. PIWIL2 has been described as being expressed in the germline during early spermatogenesis [40], [41]. However, PIWIL1 is expressed after birth in pachytene spermatocytes and spermatids and has been posited to act in translational control in the latest stages of spermatogenesis [25]. There is further evidence that PIWIL2 has essential roles in the initial phases of spermatogenesis: transposon silencing in fetal gonocytes [42], germline stem cell self-renewal [40] and early prophase of meiosis [26] in mammalian testis. Furthermore, PIWIL2 has been implicated in translational regulation of many genes during early spermatogenesis since it binds piRNAs and mRNAs [40], [43].

TDRD1 interacts directly with both PIWIL2 and PIWIL1 [44]. Although it does not affect the ability of PIWI proteins to associate with piRNAs in embryonic testes, it ensures the entry of correct transcripts into the normal piRNA pool [28].

The importance of *PIWIL2* and *TDRD1* in the efficient production of mature spermatocytes was previously reported in a model system using homozygous knock-out mice [26]–[28]. Interestingly, both recombinant mouse models revealed a common phenotype: a defect in early prophase of the first meiosis in the spermatogenesis resulting in sterility. Concordantly, our study reveals a remarkable and significant negative correlation between *PIWIL2* and *TDRD1* methylation and the number of cells from the earliest steps of spermatogenesis, spermatogoniae and spermatocytes. Although still significant, the degree of correlation was lower for postmeiotic germ cells, suggesting a weaker linear relationship between *PIWIL2* and *TDRD1* methylation and the latest stages of the spermatogenic process. The number of germ cells was positively correlated with *PIWIL2* and *TDRD1* expression in the whole tissue and with *PIWIL2* and *TDRD1* expression per cell. Taken together, these results suggest the involvement of *PIWIL2* and *TDRD1* in the human germ cell development process. We suggest that DNA hypermethylation in the promoter regions of *PIWIL2* and *TDRD1* leads to the transcriptional repression of these genes contributing to spermatogenic derangement.

Moreover, as PIWIL2 and TDRD1 physically and functionally interact in the biogenesis of piRNAs, a crucial role of these 26–31 nt small RNAs in spermatogenesis may be suspected. We identified a downregulation of mature piRNAs in SpF samples, similarly to what was described in fetal germ cells of the Mili null model [42]. The most immediate functional consequence of piRNA depletion is a derepression of repetitive elements [45]–[47]. Whether this leads directly to maturation arrest in spermatogenesis or additional functions of piRNAs and whether associated complexes contribute to the severe phenotype is currently being investigated. The repression of *PIWIL2* and *TDRD1* gene expression in the severe spermatogenic defects examined in this study, leads us to speculate that the molecular alterations affecting piRNAs and their machinery are involved in human infertility.

The aberrant methylation and expression of PIWI-family genes has the ability to provoke methylation changes of additional loci. Genetic and molecular characterization identified interactions between methyltransferases and piRNA pathway members. The PIWI/DNMT3L complex targets genomic loci, sequence-guided by small RNAs [48]. *DNMT3L* [48] as well as *PIWIL2* [49] and *TDRD1* [28] null models revealed a loss of methylation at LINE-1 and intracisternal A-particle (IAP) transposons, leading to reactivation of repetitive elements that contribute to meiotic arrest and male infertility. Consistently, we detected several SpF samples with hypomethylated LINE-1 sequences, suggesting that reactivation of transposons also participates in the human spermatogenic failure. The activation of retrotransposons affects meiotic and premeiotic germ cells, but not the later stages of spermatogenesis. Interestingly, LINE-1 methylation was only affected in sgMF and scMF samples, but not in rsMF, where the number of spermatocytes was similar to that in normal testis. This is consistent with the assumption that meiotic spermatocytes are protected against retrotransposons.

In addition to repetitive elements, single genomic loci are also targeted by PIWI complexes sequence-guided by piRNAs [50]. Here, we identified seven differentially methylated genes in SpF samples with complementary sequences to piRNAs. Taking into account that piRNA-guided binding of the PIWI complex has the ability to alter DNA methylation, we hypothesize that the differentially methylated promoters containing piRNAs binding sites are directly affected by the altered expression of *PIWIL2* and *TDRD1* in SpF.

In summary, we identified not only an aberrant DNA methylation profile at CpG sites in male infertility of testicular origin, but also DNA methylation changes in germline-specific genes, in particular *PIWIL2* and *TDRD1,* with functional consequences such as loss of DNA methylation in repetitive elements and a defective production of piRNAs. Therefore, we propose that DNA methylation, at least that affecting *PIWIL2/TDRD1*, plays a role in the control of human spermatogenic gene expression, and this process critically contributes to a successful germ cell development.

## Supporting Information

Figure S1 **Bisulfite sequencing of** ***PIWIL1*** **and** ***PIWIL2*** **in testis with conserved spermatogenesis (CS), maturation failure at the spermatocyte (scMF) or at the round spermatid (rsMF) stage, and with Sertoli cell-only syndrome (SCO).** Black and white squares indicate CpG methylation and unmethylated sites, respectively. Sample numbers are indicated.
(PDF)

Figure S2 **Bisulfite sequencing of** ***TDRD1*** **and** ***TDRD9*** **in testis with conserved spermatogenesis (CS), maturation failure at the spermatocyte (scMF) or at the round spermatid (rsMF) stage, and with Sertoli cell-only syndrome (SCO).** Black and white squares indicate CpG methylation and unmethylated sites, respectively. Sample numbers are indicated.
(PDF)

Table S1 Primer sequences and locations.
(PDF)

Table S2 633 differentially methylated CpG sites in SpF relative to normal testis tissue.
(PDF)

Table S3 Gene ontology analysis of differentially methylated genes in SpF.
(PDF)

Table S4 Differentially methylated gene promoters overlapping piRNAs.
(PDF)
