# Supplementary figures and images for: Epigenetic Disruption of the PIWI Pathway in Human Spermatogenic Disorders
Source: PLoS One. 2012 Oct 24;7(10):e47892. doi: 10.1371/journal.pone.0047892 (PMC3480440; doi:10.1371/journal.pone.0047892)

Figure S1

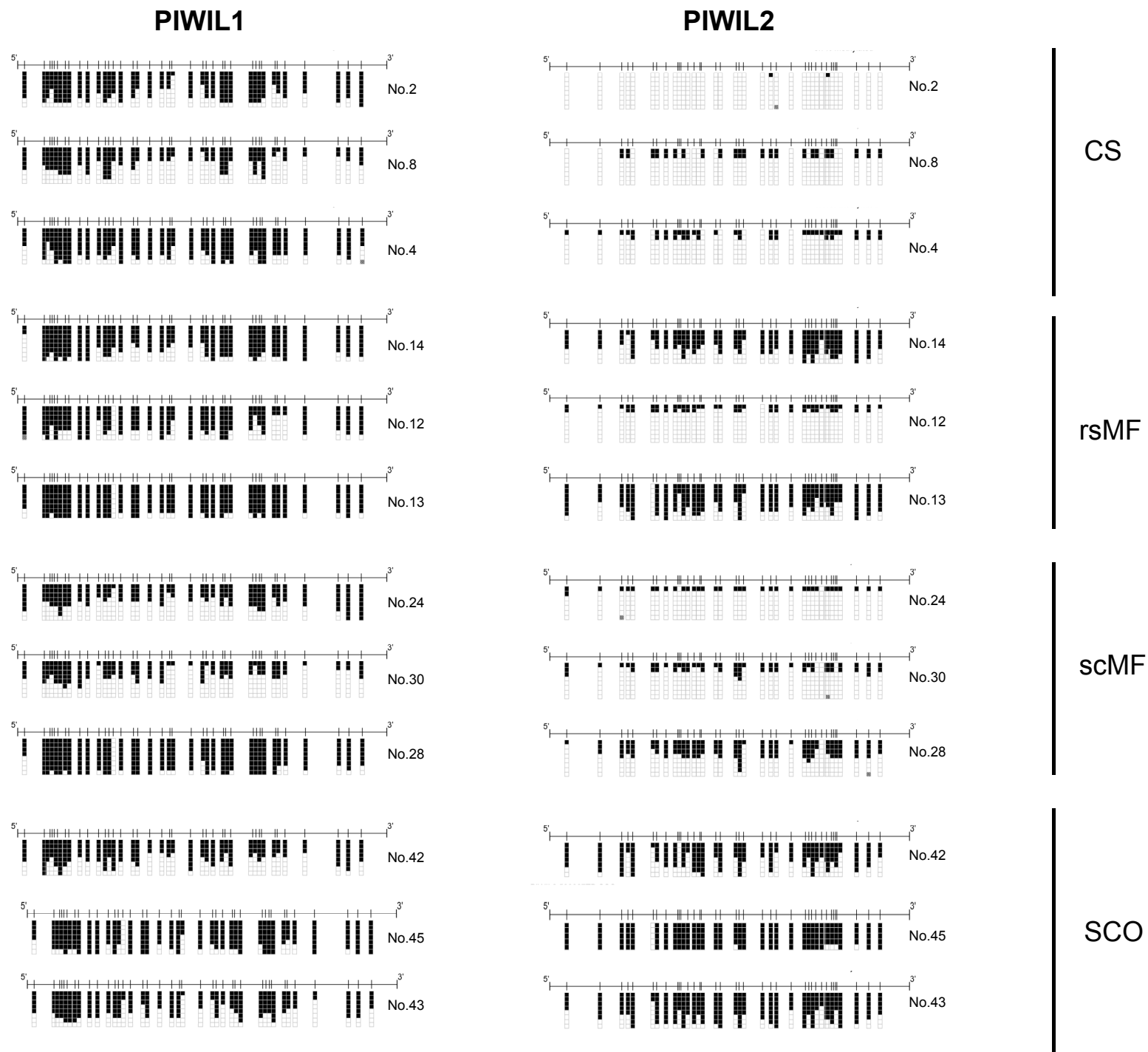

Supplement: Figure S1 — Bisulfite sequencing of PIWIL1 and PIWIL2 in testis with conserved spermatogenesis (CS), maturation failure at the spermatocyte (scMF) or at the round spermatid (rsMF) stage, and with Sertoli cell-only syndrome (SCO). Black and white squares indicate CpG methylation and unmethylated sites, respectively. Sample numbers are indicated. (PDF) [file pone.0047892.s001.pdf]

Figure S1

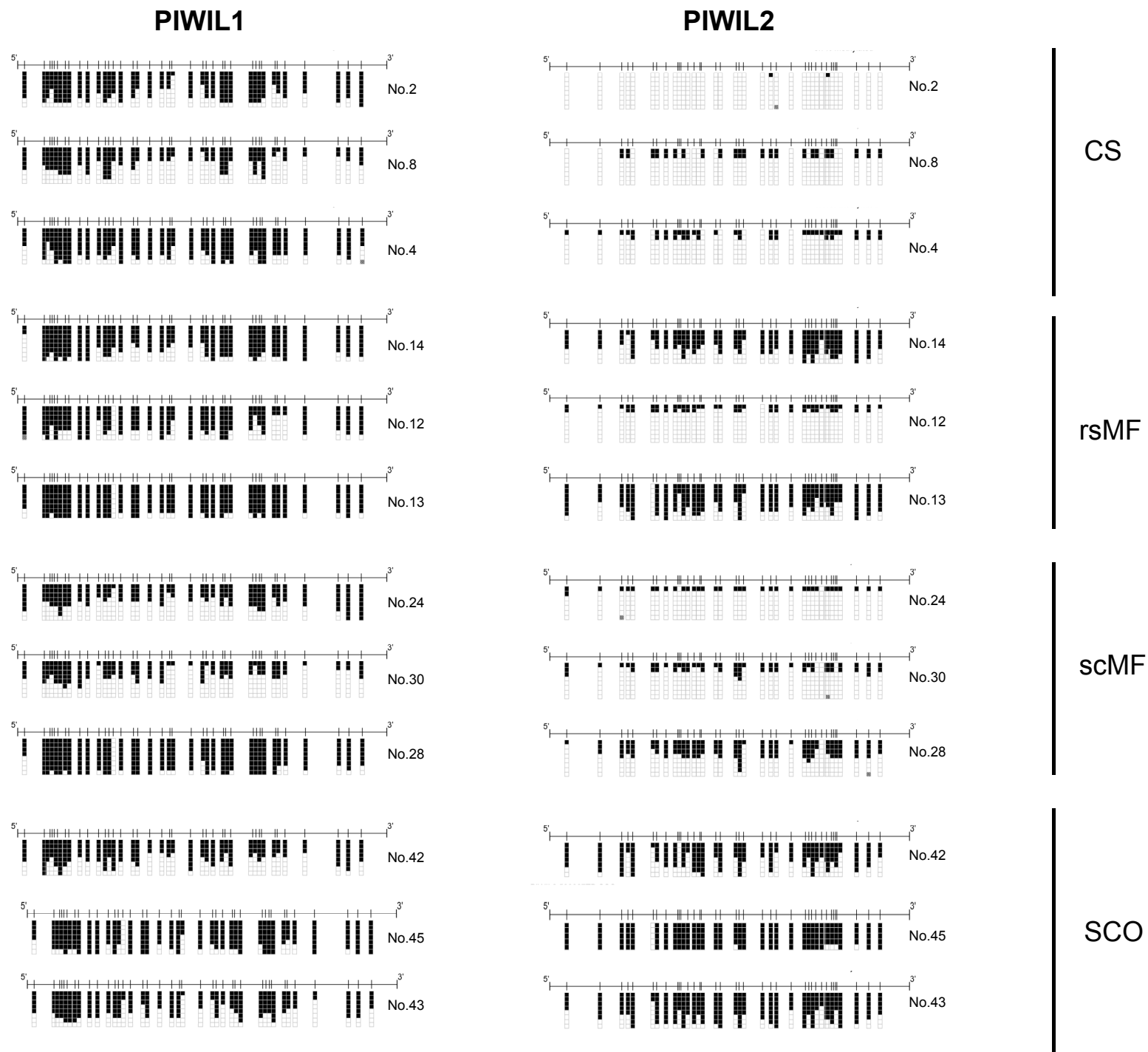

Supplement: Figure S2 — Bisulfite sequencing of TDRD1 and TDRD9 in testis with conserved spermatogenesis (CS), maturation failure at the spermatocyte (scMF) or at the round spermatid (rsMF) stage, and with Sertoli cell-only syndrome (SCO). Black and white squares indicate CpG methylation and unmethylated sites, respectively. Sample numbers are indicated. (PDF) [file pone.0047892.s002.pdf]
