# Supplementary material for: Epigenetic Disruption of the PIWI Pathway in Human Spermatogenic Disorders
Source: PLoS One. 2012 Oct 24;7(10):e47892. doi: 10.1371/journal.pone.0047892 (PMC3480440; doi:10.1371/journal.pone.0047892)
Supplement: Table S1 — Primer sequences and locations. (PDF) [file pone.0047892.s003.pdf]

Supplementary table S1: Primer sequences and locations

Gene	BS-seq (FF)	BS-seq (RV)	Location	Size	Start	Stop	TSS	TSS distance (5')	TSS distance (3')
PIWIL1	GTTTGCGGGGTAGTTAATGAG	ACCTAACACCTCAACCTAACCC	chr12:130,822,386-130,822,640	255	130,822,386	130,822,640	130822433	-47	207
PIWIL2	TATAGGGAATTTGTTGGAAAGG	CCCTACCAAAAATTCTATCTCCT	chr8:22,132,691-22,133,201	511	22,132,691	22,133,201	22132810	-119	391
TDRD1	TTGGAAAATTTTGGAAGATTTT	ACAAAATTCCAAATCTCCTCTC	chr10:115,938,773-115,939,337	565	115,938,773	115,939,337	115939029	-256	308
TDRD9	ATATATAGGGGTTGTTAYGGAA	TACACCTCCTCCCTAACC	chr14:104,394,513-104,394,971	459	104,394,513	104,394,971	104394817	-304	154

Gene	Pyro-seq (FF)	Pyro-seq (RV)	Sequencing primer	Size	Start	TSS	TSS distance (5')
PIWIL2	ATGGGTTAATTAGATAGTTTGTTTTTGTGA	AACCCACATACTCCAAAACCAATTTC	AATTAGATAGTTTGTTTTTGTGAA	106	22,132,759	22132810	-51
TDRD1	AAGGAATTTTTTGAGTTTGTAATTAGAGTA	ATACAAACCCTCTCCCTCCCCTATA	TTGTAATTAGAGTATAAGTTGTTT	296	115,938,961	115939029	-68

BS-seq (bisulphite-sequencing), Pyro-seq (pyro-sequencing), FF (forward primer), RV (reverse primer), TSS (transcription start site)
