# Supplementary material for: Epigenetic Disruption of the PIWI Pathway in Human Spermatogenic Disorders
Source: PLoS One. 2012 Oct 24;7(10):e47892. doi: 10.1371/journal.pone.0047892 (PMC3480440; doi:10.1371/journal.pone.0047892)
Supplement: Table S2 — 633 differentially methylated CpG sites in SpF relative to normal testis tissue. (PDF) [file pone.0047892.s004.pdf]

Supplementary table S2: 633 differentially methylated CpG sites in SpF relative to normal testis tissue.

Gene symbol	Chr.	Position	CpG island	Probe ID	Direction
NHLH2	1	116186057	FALSE	cg22427279	Hypermethylated
IQCH	15	65325500	FALSE	cg22940988	Hypermethylated
INSL4	9	5221360	FALSE	cg19297688	Hypermethylated
CHST11	12	103370411	FALSE	cg26843567	Hypermethylated
PRAMEF2	1	12838902	FALSE	cg18807515	Hypermethylated
XAGE5	X	52857461	TRUE	cg25993152	Hypermethylated
KCNAB2	1	6008459	TRUE	cg13614083	Hypermethylated
C6orf118	6	165643786	FALSE	cg05799317	Hypermethylated
CRYBA4	22	25348010	FALSE	cg26822175	Hypermethylated
AQP12A	2	241280171	TRUE	cg06356454	Hypermethylated
STMN4	8	27172130	FALSE	cg02130905	Hypermethylated
SLC37A1	21	42792922	TRUE	cg17398613	Hypermethylated
MOXD1	6	132764857	TRUE	cg13603171	Hypermethylated
NGFRAP1	X	102517067	TRUE	cg00725777	Hypermethylated
TKTL2	4	164614599	TRUE	cg16413535	Hypermethylated
CLEC2A	12	9976203	FALSE	cg24820250	Hypermethylated
SLC25A16	10	69958382	TRUE	cg08620470	Hypermethylated
TCEB3C	18	42811633	FALSE	cg02432101	Hypermethylated
C8orf45	8	67948348	FALSE	cg10960626	Hypermethylated
CYP4F11	19	15906119	FALSE	cg24655310	Hypermethylated
SSX5	X	47941234	TRUE	cg23812886	Hypermethylated
KLF17	1	44356937	TRUE	cg08679985	Hypermethylated
PSG3	19	47937700	FALSE	cg07745725	Hypermethylated
CRYGN	7	150768409	FALSE	cg22830895	Hypermethylated
C20orf75	20	5982446	FALSE	cg23629496	Hypermethylated
OR12D2	6	29472397	FALSE	cg04737405	Hypermethylated
SNRPN	15	22644614	TRUE	cg22678136	Hypermethylated
MYO18B	22	24468429	TRUE	cg07654843	Hypermethylated
XAGE3	X	52913717	TRUE	cg17919471	Hypermethylated
C16orf33	16	42121	TRUE	cg14820573	Hypermethylated
CRH	8	67253306	FALSE	cg18640030	Hypermethylated
CDR1	X	139694161	FALSE	cg08214957	Hypermethylated
ENOSF1	18	703200	TRUE	cg16112050	Hypermethylated
SYCE1	10	135232535	TRUE	cg17676129	Hypermethylated
ZNF169	9	96080281	FALSE	cg15264273	Hypermethylated
TBC1D22B	6	37332194	FALSE	cg02187357	Hypermethylated
C14orf152	14	93455148	FALSE	cg20022541	Hypermethylated
C3orf14	3	62280559	FALSE	cg09473585	Hypermethylated
TCEB3C	18	42811593	FALSE	cg07008350	Hypermethylated

PF4	4	75066510	TRUE	cg15158783	Hypermethylated
IL2RA	10	6144318	TRUE	cg11733245	Hypermethylated
RAB9P1	5	104462726	FALSE	cg15453943	Hypermethylated
FAM50B	6	3794271	TRUE	cg01570885	Hypermethylated
C21orf6	21	29314090	TRUE	cg05406101	Hypermethylated
FFAR2	19	40632702	TRUE	cg15479752	Hypermethylated
TMPRSS3	21	42689155	TRUE	cg01214847	Hypermethylated
ATP1A2	1	158351403	FALSE	cg08390254	Hypermethylated
POGZ	1	149699846	FALSE	cg12647320	Hypermethylated
C10orf111	10	15180418	TRUE	cg00260778	Hypermethylated
RAB8A	19	16082719	TRUE	cg03621001	Hypermethylated
SEPT9	17	72827828	TRUE	cg03330678	Hypermethylated
SPACA1	6	88814597	TRUE	cg13334277	Hypermethylated
C12orf26	12	81275119	FALSE	cg02845923	Hypermethylated
MAP4K2	11	64328475	FALSE	cg10821722	Hypermethylated
KLK4	19	56107264	TRUE	cg10078829	Hypermethylated
USP29	19	62322503	TRUE	cg16547341	Hypermethylated
HPN	19	40223257	TRUE	cg06151964	Hypermethylated
NALP2	19	60169541	TRUE	cg16106497	Hypermethylated
USP29	19	62323044	TRUE	cg07499372	Hypermethylated
DSG3	18	27280299	TRUE	cg08555924	Hypermethylated
PSKH2	8	87151181	TRUE	cg19587887	Hypermethylated
ATP10A	15	23660297	TRUE	cg08828036	Hypermethylated
C21orf77	21	32870432	FALSE	cg13033054	Hypermethylated
KRTAP17-1	17	36725264	FALSE	cg16242770	Hypermethylated
PGLYRP1	19	51218323	TRUE	cg10862535	Hypermethylated
CCDC27	1	3659032	FALSE	cg10051054	Hypermethylated
SLN	11	107088014	FALSE	cg17971003	Hypermethylated
DIRAS3	1	68285651	TRUE	cg21808053	Hypermethylated
FLJ23322	22	35232524	TRUE	cg05475277	Hypermethylated
TAZ	X	153292481	FALSE	cg11331769	Hypermethylated
CTAG1B	X	153500553	TRUE	cg25531166	Hypermethylated
LMO3	12	16652673	FALSE	cg11511443	Hypermethylated
MAGEB6	X	26120444	FALSE	cg10127415	Hypermethylated
H2AFB3	X	154342853	TRUE	cg06532147	Hypermethylated
SNRPN	15	22674584	TRUE	cg25657700	Hypermethylated
RCN3	19	54722273	TRUE	cg03907174	Hypermethylated
MAGEA10	X	151057709	TRUE	cg19964192	Hypermethylated
HMGCL	1	24025361	TRUE	cg18888403	Hypermethylated
AMHR2	12	52102542	FALSE	cg18715299	Hypermethylated
MMP13	11	102331300	FALSE	cg13041032	Hypermethylated
DDX4	5	55069371	TRUE	cg02899723	Hypermethylated
C20orf102	20	35965510	TRUE	cg11599505	Hypermethylated
DHX8	17	38916826	TRUE	cg23347958	Hypermethylated
PARP12	7	139410242	FALSE	cg24435704	Hypermethylated
ZNF619	3	40493095	FALSE	cg21942438	Hypermethylated
CENTG1	12	56423446	FALSE	cg17611475	Hypermethylated

PEG10	7	94131716	TRUE	cg06943865	Hypermethylated
MSH4	1	76035125	TRUE	cg21438018	Hypermethylated
TCL1A	14	95250072	TRUE	cg14127336	Hypermethylated
ZNF645	X	22200841	TRUE	cg20063650	Hypermethylated
DHODH	16	70598877	TRUE	cg22381196	Hypermethylated
ZNF445	3	44494987	FALSE	cg18129786	Hypermethylated
DNAJC15	13	42494656	TRUE	cg00131557	Hypermethylated
FAM9C	X	12972572	TRUE	cg13918808	Hypermethylated
CLDN17	21	30460420	FALSE	cg13792279	Hypermethylated
SCTR	2	119998797	TRUE	cg01897036	Hypermethylated
MAGEA11	X	148604241	FALSE	cg00799727	Hypermethylated
ZNF80	3	115440593	FALSE	cg03109316	Hypermethylated
TTLL11	9	123896217	TRUE	cg27218220	Hypermethylated
KCNAB2	1	6009035	TRUE	cg19814116	Hypermethylated
LOC340602	X	51166482	TRUE	cg20931907	Hypermethylated
INSL3	19	17792995	TRUE	cg06189133	Hypermethylated
ACVRL1	12	50587901	TRUE	cg23640701	Hypermethylated
PIWIL1	12	129388239	TRUE	cg13861644	Hypermethylated
CYP2E1	10	135190730	FALSE	cg00436603	Hypermethylated
LRRC25	19	18370505	FALSE	cg08379517	Hypermethylated
PIWIL2	8	22188937	TRUE	cg18135555	Hypermethylated
C8orf78	8	125252939	TRUE	cg05412531	Hypermethylated
AP2M1	3	185374622	FALSE	cg24661752	Hypermethylated
BIRC8	19	58486766	FALSE	cg09377486	Hypermethylated
ACTL8	1	17954590	FALSE	cg19685066	Hypermethylated
MMAA	4	146778997	TRUE	cg25421002	Hypermethylated
STK31	7	23716132	TRUE	cg14898779	Hypermethylated
SLC25A2	5	140664331	TRUE	cg26783353	Hypermethylated
PTPN20B	10	48448115	TRUE	cg02222362	Hypermethylated
HPN	19	40223243	TRUE	cg24715735	Hypermethylated
MGC15763	3	16280294	FALSE	cg10579246	Hypermethylated
FLJ22746	2	224974590	TRUE	cg26154999	Hypermethylated
OLFM4	13	52501287	FALSE	cg12582008	Hypermethylated
KCNQ1	11	2447807	TRUE	cg01734338	Hypermethylated
TCEB3C	18	42811405	FALSE	cg16907024	Hypermethylated
FCGR2A	1	159741600	TRUE	cg24422489	Hypermethylated
CHM	X	85190083	FALSE	cg06340713	Hypermethylated
ZNF300	5	150264078	FALSE	cg12346881	Hypermethylated
C1orf177	1	55044265	TRUE	cg20903926	Hypermethylated
GNAS	20	56850283	TRUE	cg01355739	Hypermethylated
CASQ2	1	116112864	FALSE	cg18942631	Hypermethylated
PKDREJ	22	45038062	TRUE	cg08315277	Hypermethylated
GNAS	20	56861225	TRUE	cg17414107	Hypermethylated
FOLH1	11	49187124	TRUE	cg06980460	Hypermethylated
C9orf98	9	134745184	FALSE	cg03382797	Hypermethylated
CLTCL1	22	17660165	TRUE	cg07251788	Hypermethylated
COQ3	6	99949074	TRUE	cg24831427	Hypermethylated

SLC24A5	15	46200510	FALSE	cg01497576	Hypermethylated
MAGEB2	X	30143683	TRUE	cg17299712	Hypermethylated
IMP4	2	130817933	TRUE	cg26385743	Hypermethylated
SFRS16	19	50233032	FALSE	cg25203561	Hypermethylated
FLJ32894	12	24628518	TRUE	cg04926244	Hypermethylated
NEK3	13	51633076	FALSE	cg19524009	Hypermethylated
C19orf33	19	43486685	FALSE	cg00412772	Hypermethylated
ELAC1	18	46747757	TRUE	cg11911418	Hypermethylated
FLJ32569	1	204085874	TRUE	cg14893161	Hypermethylated
MGC50273	2	132275409	TRUE	cg25953146	Hypermethylated
GNAS	20	56860330	TRUE	cg03606258	Hypermethylated
C8orf78	8	125252957	TRUE	cg00551244	Hypermethylated
TFAP2E	1	35811101	TRUE	cg11835197	Hypermethylated
GNAS	20	56862672	TRUE	cg21988465	Hypermethylated
C20orf86	20	56236484	FALSE	cg09898548	Hypermethylated
CTHRC1	8	104453467	TRUE	cg19188612	Hypermethylated
TAAR6	6	132933198	TRUE	cg12813797	Hypermethylated
MAGEA9	X	148671471	FALSE	cg02649337	Hypermethylated
FMO2	1	169421236	FALSE	cg14721213	Hypermethylated
FOXI1	5	169465330	TRUE	cg19233472	Hypermethylated
WDR45L	17	78200236	TRUE	cg10281770	Hypermethylated
IL22	12	66933282	FALSE	cg26333641	Hypermethylated
CLDN8	21	30509994	FALSE	cg04052038	Hypermethylated
GPR1	2	206787397	TRUE	cg19132372	Hypermethylated
PLG	6	161043793	FALSE	cg09087901	Hypermethylated
SPATA16	3	174341716	TRUE	cg01216369	Hypermethylated
PSG1	19	48076917	FALSE	cg25839766	Hypermethylated
USP52	12	55015247	TRUE	cg26265187	Hypermethylated
PSMC5	17	59257059	FALSE	cg07117700	Hypermethylated
FAM9A	X	8729528	TRUE	cg00600110	Hypermethylated
CCDC19	1	158135847	TRUE	cg09451092	Hypermethylated
KRT6E	12	51154060	FALSE	cg08136806	Hypermethylated
FRG1	4	191099164	TRUE	cg01737532	Hypermethylated
HAT1	2	172486678	TRUE	cg24611092	Hypermethylated
ZNF22	10	44815441	FALSE	cg01614759	Hypermethylated
SLC35D2	9	98186585	TRUE	cg22324153	Hypermethylated
MGC40168	1	6219715	TRUE	cg06940574	Hypermethylated
TM4SF19	3	197549966	FALSE	cg05445326	Hypermethylated
CTNNA3	10	69126313	FALSE	cg00132141	Hypermethylated
GCN5L2	17	37527341	TRUE	cg04278905	Hypermethylated
TEX13B	X	107112286	FALSE	cg10857345	Hypermethylated
OSCAR	19	59297454	TRUE	cg21098323	Hypermethylated
NNAT	20	35583475	TRUE	cg10642330	Hypermethylated
C8orf31	8	144192081	FALSE	cg04612566	Hypermethylated
MBP	18	72974410	TRUE	cg12555907	Hypermethylated
VTI1B	14	67212286	TRUE	cg04269351	Hypermethylated
ZIM2	19	61998893	TRUE	cg06244906	Hypermethylated

SEMA3B	3	50289658	TRUE	cg13550608	Hypermethylated
UBE2U	1	64442187	TRUE	cg10753073	Hypermethylated
GNAS	20	56860144	TRUE	cg15160445	Hypermethylated
KRTCAP3	2	27519047	TRUE	cg11618577	Hypermethylated
HLA-DRB5	6	32606582	FALSE	cg10746737	Hypermethylated
IL13RA2	X	114159571	FALSE	cg00488364	Hypermethylated
OR2A4	6	132064183	FALSE	cg17026542	Hypermethylated
HDAC11	3	13497740	TRUE	cg05446471	Hypermethylated
IL20RA	6	137408015	TRUE	cg22487322	Hypermethylated
TPCN2	11	68572025	TRUE	cg22484980	Hypermethylated
BRDT	1	92187310	TRUE	cg14732540	Hypermethylated
ATP9A	20	49819573	FALSE	cg05851042	Hypermethylated
LOC201164	17	17050295	TRUE	cg05590257	Hypermethylated
ATP10A	15	23523421	TRUE	cg12582965	Hypermethylated
C11orf47	11	6474863	TRUE	cg25368651	Hypermethylated
SNRPN	15	22674642	TRUE	cg24993443	Hypermethylated
RAI16	8	22002058	FALSE	cg24471555	Hypermethylated
HPS1	10	100196915	TRUE	cg20340596	Hypermethylated
DNAH8	6	38798856	TRUE	cg24760768	Hypermethylated
CREBBP	16	3871886	TRUE	cg03264209	Hypermethylated
MAGEA2	X	151672941	TRUE	cg01743008	Hypermethylated
TCEB3C	18	42811817	FALSE	cg08008233	Hypermethylated
DPM1	20	49009813	TRUE	cg16332224	Hypermethylated
GALNTL5	7	151284387	FALSE	cg11091262	Hypermethylated
DKFZP779L1558	12	28301371	FALSE	cg17489451	Hypermethylated
UBD	6	29636098	FALSE	cg07326586	Hypermethylated
SFN	1	27062374	TRUE	cg03421300	Hypermethylated
RABGEF1	7	65842232	FALSE	cg18884741	Hypermethylated
	3	144378065	TRUE	cg10883352	Hypermethylated
MGC3207	19	13736430	TRUE	cg20555481	Hypermethylated
BCL2	18	59054814	TRUE	cg08223235	Hypermethylated
MAPK13	6	36205670	FALSE	cg05859264	Hypermethylated
IKBKAP	9	110737466	FALSE	cg25018881	Hypermethylated
ZNF678	1	225817515	TRUE	cg26683023	Hypermethylated
TM4SF19	3	197549503	FALSE	cg05556202	Hypermethylated
GPRC5D	12	12994922	FALSE	cg12800145	Hypermethylated
NTSR1	20	60811330	TRUE	cg14871138	Hypermethylated
FRK	6	116488302	FALSE	cg16176600	Hypermethylated
C14orf58	14	75115101	TRUE	cg04001333	Hypermethylated
RAB24	5	176664728	FALSE	cg13284426	Hypermethylated
NPFFR2	4	73116145	FALSE	cg03017946	Hypermethylated
SMPD3	16	67040695	TRUE	cg17217677	Hypermethylated
GPATC1	19	38263092	FALSE	cg10108208	Hypermethylated
SPAG7	17	4813327	TRUE	cg12815142	Hypermethylated
CHM	X	85189965	FALSE	cg25488547	Hypermethylated
GPR39	2	132891105	TRUE	cg07785936	Hypermethylated
CLEC2A	12	9976441	FALSE	cg27190239	Hypermethylated

ZBTB40	1	22650259	TRUE	cg26870337	Hypermethylated
GPKOW	X	48867638	TRUE	cg17705081	Hypermethylated
HIST1H2AA	6	25834576	TRUE	cg01307730	Hypermethylated
CNGA1	4	47650768	TRUE	cg19000186	Hypermethylated
KRTAP19-5	21	30797319	FALSE	cg07374637	Hypermethylated
GAS6	13	113545690	TRUE	cg14358743	Hypermethylated
CLPB	11	71823904	TRUE	cg05812599	Hypermethylated
PDF	16	67922724	TRUE	cg12897458	Hypermethylated
MAGEC1	X	140819380	TRUE	cg11110686	Hypermethylated
OXA1L	14	22304812	TRUE	cg20782689	Hypermethylated
HOXD3	2	176735887	TRUE	cg18702197	Hypermethylated
GPR45	2	105224921	TRUE	cg22253945	Hypermethylated
PROCA1	17	24063650	TRUE	cg04578090	Hypermethylated
DNAJC15	13	42494577	TRUE	cg11679069	Hypermethylated
GLRA2	X	14457801	FALSE	cg05206587	Hypermethylated
PNMA6A	X	151990896	TRUE	cg06904813	Hypermethylated
VCY	Y	14677478	TRUE	cg15812957	Hypermethylated
FKBP4	12	2773051	TRUE	cg11518240	Hypermethylated
TMPRSS3	21	42689424	TRUE	cg25608949	Hypermethylated
BOK	2	242146552	TRUE	cg00603172	Hypermethylated
COPA	1	158580685	FALSE	cg08015496	Hypermethylated
HNF4A	20	42418292	TRUE	cg19717150	Hypermethylated
KLF17	1	44357517	TRUE	cg18202456	Hypermethylated
UBD	6	29635984	FALSE	cg15320474	Hypermethylated
GGTLA4	20	23917432	TRUE	cg13424446	Hypermethylated
OR2A4	6	132064978	FALSE	cg11884699	Hypermethylated
ACP1	2	255872	FALSE	cg27226618	Hypermethylated
RB1	13	47793479	TRUE	cg07880715	Hypermethylated
SLC13A4	7	135063389	FALSE	cg02005755	Hypermethylated
TMEM43	3	14140187	FALSE	cg14375111	Hypermethylated
FANCG	9	35070812	TRUE	cg05293216	Hypermethylated
AMPD3	11	10427725	TRUE	cg08035082	Hypermethylated
PDE4C	19	18205322	TRUE	cg13899108	Hypermethylated
C6orf55	6	142508736	FALSE	cg10035272	Hypermethylated
C9orf41	9	76834070	TRUE	cg18168989	Hypermethylated
TOLLIP	11	1288312	TRUE	cg22424444	Hypermethylated
ASZ1	7	116854652	TRUE	cg16540704	Hypermethylated
NSUN3	3	95263136	TRUE	cg04032566	Hypermethylated
C15orf24	15	32182832	FALSE	cg18099844	Hypermethylated
ZIM2	19	61998659	TRUE	cg16519742	Hypermethylated
MAGEA4	X	150831952	TRUE	cg22497867	Hypermethylated
C21orf56	21	46429602	TRUE	cg10296238	Hypermethylated
LOC348840	3	199293030	TRUE	cg02141570	Hypermethylated
ILDR1	3	123224320	TRUE	cg04059863	Hypermethylated
DUS4L	7	106990684	FALSE	cg19770955	Hypermethylated
PRDM2	1	13902462	FALSE	cg25402049	Hypermethylated
BCL2	18	59055308	TRUE	cg25059899	Hypermethylated

CYP26C1	10	94809792	TRUE	cg26404725	Hypermethylated
ZCCHC13	X	73440678	TRUE	cg04211807	Hypermethylated
SEMA6B	19	4509119	TRUE	cg10052840	Hypermethylated
FLJ25421	22	38619484	TRUE	cg08245789	Hypermethylated
TCEB3B	18	42815723	TRUE	cg24883732	Hypermethylated
DMRTC1	X	72014324	FALSE	cg25268283	Hypermethylated
TMEM45B	11	129190572	FALSE	cg18913951	Hypermethylated
DHX40	17	54997457	TRUE	cg18062196	Hypermethylated
PLAGL1	6	144371522	TRUE	cg17895149	Hypermethylated
C14orf11	14	34079243	TRUE	cg24276491	Hypermethylated
AKR1C1	10	4994250	FALSE	cg07639198	Hypermethylated
PTPN20B	10	48448112	TRUE	cg16192575	Hypermethylated
UCN	2	27384674	TRUE	cg04527918	Hypermethylated
SETBP1	18	40535761	FALSE	cg00037763	Hypermethylated
GLRX2	1	191341814	FALSE	cg15361231	Hypermethylated
TCEB3B	18	42816134	TRUE	cg20879768	Hypermethylated
KEAP1	19	10476199	TRUE	cg03754063	Hypermethylated
PAGE1	X	49347342	TRUE	cg23937047	Hypermethylated
HIST1H1T	6	26216314	TRUE	cg19515446	Hypermethylated
LOC223075	7	31523600	TRUE	cg25650811	Hypermethylated
ACTL7B	9	110657950	TRUE	cg05156613	Hypermethylated
ZFYVE19	15	38885547	FALSE	cg03012280	Hypermethylated
GRB10	7	50817425	TRUE	cg08835688	Hypermethylated
SGCB	4	52599782	TRUE	cg12489960	Hypermethylated
MGC27016	4	155921670	TRUE	cg02992596	Hypermethylated
SLC6A14	X	115482720	FALSE	cg00894577	Hypermethylated
HEPH	X	65299801	FALSE	cg08021299	Hypermethylated
MAN2C1	15	73448502	TRUE	cg04008455	Hypermethylated
ARD1A	X	152854847	TRUE	cg00434413	Hypermethylated
MAGEA3	X	151688792	TRUE	cg16390856	Hypermethylated
MAGEA1	X	152139132	FALSE	cg23776892	Hypermethylated
ADCY2	5	7448318	TRUE	cg04907257	Hypermethylated
INDOL1	8	39910381	FALSE	cg16957569	Hypomethylated
DCN	12	90100572	FALSE	cg04088433	Hypomethylated
IKBKE	1	204710788	FALSE	cg22577136	Hypomethylated
CCL26	7	75257240	FALSE	cg05556717	Hypomethylated
ABCA8	17	64462140	FALSE	cg21660392	Hypomethylated
OBP2A	9	137577873	FALSE	cg15092802	Hypomethylated
REG1B	2	79168253	FALSE	cg07841014	Hypomethylated
C1R	12	7136253	FALSE	cg16236875	Hypomethylated
FLJ39501	19	15479559	FALSE	cg14851685	Hypomethylated
CYP4F3	19	15612167	FALSE	cg16377880	Hypomethylated
C16orf30	16	1522582	FALSE	cg17706173	Hypomethylated
MARK2	11	63412666	FALSE	cg06204948	Hypomethylated
TSPAN8	12	69838455	FALSE	cg15684563	Hypomethylated
THBS3	1	153443575	FALSE	cg25912717	Hypomethylated
SH3PX3	15	73728631	FALSE	cg27635271	Hypomethylated

TMEM10	10	98109069	FALSE	cg06825166	Hypomethylated
BST2	19	17377470	FALSE	cg01254505	Hypomethylated
NOTCH4	6	32299818	FALSE	cg14700707	Hypomethylated
BRD4	19	15252927	TRUE	cg08044694	Hypomethylated
MGMT	10	131302691	TRUE	cg18488970	Hypomethylated
CLDN20	6	155626401	FALSE	cg17306637	Hypomethylated
HYMAI	6	144376426	FALSE	cg08256027	Hypomethylated
GPR156	3	121445898	FALSE	cg19093820	Hypomethylated
RRH	4	110967856	FALSE	cg19428735	Hypomethylated
PROK1	1	110795047	FALSE	cg18434152	Hypomethylated
MYO1F	19	8549540	FALSE	cg16504798	Hypomethylated
AQP10	1	152560106	FALSE	cg20713492	Hypomethylated
CST9L	20	23496991	FALSE	cg15210427	Hypomethylated
PDGFRB	5	149515888	TRUE	cg12727795	Hypomethylated
S100A16	1	151851544	FALSE	cg23499956	Hypomethylated
TNKS1BP1	11	56845942	FALSE	cg24855780	Hypomethylated
CCL18	17	31415591	FALSE	cg08214029	Hypomethylated
ID3	1	23758594	TRUE	cg22258437	Hypomethylated
AQP5	12	48640218	FALSE	cg19220825	Hypomethylated
HSD3B7	16	30904131	FALSE	cg10917602	Hypomethylated
LGALS8	1	234746847	FALSE	cg03082060	Hypomethylated
PARVG	22	42906788	FALSE	cg26861460	Hypomethylated
TESC	12	116022841	FALSE	cg06750167	Hypomethylated
CLEC4F	2	70901157	FALSE	cg21148892	Hypomethylated
UBE1L	3	49826443	FALSE	cg09874127	Hypomethylated
CA9	9	35663909	FALSE	cg19257550	Hypomethylated
C1orf182	1	154572288	FALSE	cg24042452	Hypomethylated
TIMD4	5	156322277	FALSE	cg18994063	Hypomethylated
KLK1	19	56019168	FALSE	cg26415633	Hypomethylated
CLEC3B	3	45041975	FALSE	cg21057494	Hypomethylated
SLC6A12	12	192511	FALSE	cg20938359	Hypomethylated
C8orf4	8	40129600	FALSE	cg17939444	Hypomethylated
RSAD2	2	6935238	FALSE	cg18201077	Hypomethylated
FKBP9L	7	55724354	TRUE	cg11808544	Hypomethylated
OSBPL7	17	43254772	FALSE	cg09911755	Hypomethylated
CLEC10A	17	6924715	FALSE	cg01472101	Hypomethylated
PRG1	10	70517405	FALSE	cg27208307	Hypomethylated
ARPM2	1	2927859	FALSE	cg04716261	Hypomethylated
C4orf18	4	159313291	FALSE	cg25841987	Hypomethylated
CARD14	17	75766923	TRUE	cg05187322	Hypomethylated
TNNI3	19	60360903	FALSE	cg03097995	Hypomethylated
FLJ43582	8	38505134	FALSE	cg23843812	Hypomethylated
SPARCL1	4	88669530	FALSE	cg19466563	Hypomethylated
TNFRSF4	1	1138954	TRUE	cg22335801	Hypomethylated
C10orf10	10	44793718	FALSE	cg04444771	Hypomethylated
TFF3	21	42608574	FALSE	cg04806409	Hypomethylated
TRPV6	7	142293853	FALSE	cg16752583	Hypomethylated

EMP1	12	13240991	FALSE	cg14409083	Hypomethylated
MRGPRF	11	68536712	FALSE	cg22933847	Hypomethylated
SLC17A4	6	25862169	FALSE	cg21627181	Hypomethylated
BCMO1	16	79829782	FALSE	cg22947000	Hypomethylated
HFE2	1	144124550	FALSE	cg06589885	Hypomethylated
CLEC2B	12	9913702	FALSE	cg17475456	Hypomethylated
XPNPEP2	X	128700621	FALSE	cg23026995	Hypomethylated
IL17RC	3	9934128	FALSE	cg07705835	Hypomethylated
FLJ20273	4	40212781	TRUE	cg01704534	Hypomethylated
MGC18079	16	27806987	FALSE	cg26062370	Hypomethylated
NCALD	8	102872413	FALSE	cg17084151	Hypomethylated
FLJ11000	7	134483310	FALSE	cg13120814	Hypomethylated
RANBP1	22	18483649	TRUE	cg11594228	Hypomethylated
SNX26	19	40958680	TRUE	cg03007010	Hypomethylated
IL19	1	205037818	FALSE	cg12380764	Hypomethylated
TRIM54	2	27358950	FALSE	cg25218351	Hypomethylated
ITGAD	16	31312602	FALSE	cg02164442	Hypomethylated
NFAM1	22	41158069	FALSE	cg17568996	Hypomethylated
INHBE	12	56135537	FALSE	cg20664201	Hypomethylated
TNS1	2	218516897	FALSE	cg18328334	Hypomethylated
GPR24	22	39405619	FALSE	cg21342728	Hypomethylated
MR1	1	179269292	FALSE	cg01040850	Hypomethylated
FLJ43582	8	38505392	FALSE	cg11653466	Hypomethylated
NYX	X	41191215	FALSE	cg18230216	Hypomethylated
SDSL	12	112344252	FALSE	cg05149586	Hypomethylated
CSF1R	5	149472673	FALSE	cg07260017	Hypomethylated
GDF3	12	7739914	FALSE	cg22956254	Hypomethylated
OIT3	10	74323455	FALSE	cg05998983	Hypomethylated
RUFY3	4	71806586	FALSE	cg06059810	Hypomethylated
FLRT3	20	14266410	FALSE	cg26082838	Hypomethylated
IL31RA	5	55185257	FALSE	cg15091410	Hypomethylated
RDH12	14	67259210	FALSE	cg04394967	Hypomethylated
TMEM86B	19	60432000	FALSE	cg26457013	Hypomethylated
C20orf75	20	5983139	FALSE	cg01869233	Hypomethylated
CTSS	1	149004797	FALSE	cg08578023	Hypomethylated
ACAT2	6	160102428	TRUE	cg15298323	Hypomethylated
IL1B	2	113310846	FALSE	cg07935264	Hypomethylated
SERPINE1	7	100557134	FALSE	cg02273392	Hypomethylated
SNCG	10	88708373	TRUE	cg21012874	Hypomethylated
CDCP2	1	54392033	TRUE	cg26185508	Hypomethylated
ELSPBP1	19	53189549	FALSE	cg08981777	Hypomethylated
SLC25A18	22	16423043	FALSE	cg02555579	Hypomethylated
PSPN	19	6327576	FALSE	cg10280342	Hypomethylated
WFDC13	20	43764255	FALSE	cg09874776	Hypomethylated
WFDC10A	20	43691798	FALSE	cg02605634	Hypomethylated
HTR2A	13	46367655	FALSE	cg00308665	Hypomethylated
NALP10	11	7941549	FALSE	cg18484189	Hypomethylated

MVP	16	29732100	FALSE	cg00334507	Hypomethylated
OR5P2	11	7774741	FALSE	cg13410437	Hypomethylated
DKK4	8	42354132	FALSE	cg21209091	Hypomethylated
WFDC10A	20	43691851	FALSE	cg11953868	Hypomethylated
CTRL	16	66523368	FALSE	cg04413148	Hypomethylated
HAS1	19	56919850	TRUE	cg13300756	Hypomethylated
CX3CL1	16	55964456	FALSE	cg15195412	Hypomethylated
SH3TC2	5	148422887	FALSE	cg01965939	Hypomethylated
MID1	X	10761548	FALSE	cg20244073	Hypomethylated
PAQR7	1	26070442	FALSE	cg15662251	Hypomethylated
SLC2A5	1	9052074	FALSE	cg03679305	Hypomethylated
FCRLM2	1	159959712	FALSE	cg27495845	Hypomethylated
BTN3A2	6	26472772	FALSE	cg14345882	Hypomethylated
FLJ35784	19	7468351	FALSE	cg16732901	Hypomethylated
ACVR1	2	158403404	FALSE	cg09499849	Hypomethylated
UGT2B17	4	69116277	FALSE	cg19481811	Hypomethylated
KIAA1822L	1	220787998	FALSE	cg20582779	Hypomethylated
KLHL6	3	184756501	FALSE	cg20398399	Hypomethylated
BTK	X	100527943	FALSE	cg03791917	Hypomethylated
FLJ35785	15	20806668	FALSE	cg06907544	Hypomethylated
TNFSF15	9	116608409	FALSE	cg10791260	Hypomethylated
SEPP1	5	42847709	FALSE	cg04502814	Hypomethylated
CLIC2	X	154217162	FALSE	cg21248478	Hypomethylated
C20orf160	20	30062162	FALSE	cg24691461	Hypomethylated
C20orf38	20	12937844	FALSE	cg25125453	Hypomethylated
FLJ37538	7	99920258	FALSE	cg08626653	Hypomethylated
EPHX1	1	224079633	FALSE	cg03459809	Hypomethylated
TDG	12	102883862	TRUE	cg03923277	Hypomethylated
PLA2G1B	12	119250606	FALSE	cg16396488	Hypomethylated
FYB	5	39255455	FALSE	cg13703437	Hypomethylated
POT1	7	124325896	FALSE	cg21792432	Hypomethylated
DMBT1	10	124310054	FALSE	cg11976790	Hypomethylated
FLJ35725	4	8491500	TRUE	cg14430151	Hypomethylated
RDH5	12	54400422	FALSE	cg02192520	Hypomethylated
SLC23A1	5	138747273	FALSE	cg25374813	Hypomethylated
OSBPL7	17	43254387	FALSE	cg23452458	Hypomethylated
BGN	X	152414100	TRUE	cg19618706	Hypomethylated
NR1H4	12	99391889	FALSE	cg23256150	Hypomethylated
PHKG1	7	56128181	FALSE	cg19759064	Hypomethylated
C1R	12	7137031	FALSE	cg15776355	Hypomethylated
DDT	22	22652308	TRUE	cg24599739	Hypomethylated
NFKBIB	19	44081059	FALSE	cg27442349	Hypomethylated
SCN2A2	2	165858646	FALSE	cg08347960	Hypomethylated
LIX1	5	96504251	FALSE	cg06213287	Hypomethylated
SOX15	17	7434875	FALSE	cg13098960	Hypomethylated
RHO	3	130729613	FALSE	cg13180098	Hypomethylated
KRT6A	12	51174747	FALSE	cg11471401	Hypomethylated

PLEKHA6	1	202595930	FALSE	cg21581873	Hypomethylated
PRTN3	19	790689	FALSE	cg00615241	Hypomethylated
PAX1	20	21627525	FALSE	cg20907471	Hypomethylated
TSPAN1	1	46418284	FALSE	cg26294850	Hypomethylated
DAND5	19	12941409	FALSE	cg15177917	Hypomethylated
CEACAM4	19	46825249	FALSE	cg10237469	Hypomethylated
RIN1	11	65860501	FALSE	cg05998426	Hypomethylated
PRAMEF1	1	12773517	FALSE	cg05372736	Hypomethylated
PHACTR4	1	28637110	FALSE	cg08123074	Hypomethylated
P8	16	28458120	FALSE	cg15149645	Hypomethylated
UPK3B	7	75977406	FALSE	cg22995176	Hypomethylated
SLC2A5	1	9052378	FALSE	cg24480859	Hypomethylated
ELSPBP1	19	53189843	FALSE	cg19404979	Hypomethylated
C3	19	6672016	FALSE	cg17612991	Hypomethylated
FAM107A	3	58538649	FALSE	cg01446393	Hypomethylated
KLK10	19	56215362	FALSE	cg06130787	Hypomethylated
IL16	15	79262693	FALSE	cg01001286	Hypomethylated
SLAMF8	1	158063058	FALSE	cg04275881	Hypomethylated
EPS8L1	19	60274447	FALSE	cg27105123	Hypomethylated
IL22RA1	1	24342151	FALSE	cg09152089	Hypomethylated
TNFRSF19	13	23042257	FALSE	cg06150803	Hypomethylated
DDR2	1	160868431	FALSE	cg17496788	Hypomethylated
ATP13A4	3	194755472	TRUE	cg23159337	Hypomethylated
CLEC3B	3	45042792	FALSE	cg06117855	Hypomethylated
LOC348174	16	68542440	TRUE	cg17091851	Hypomethylated
PDE2A	11	72062723	FALSE	cg22635155	Hypomethylated
GARNL3	9	129066841	FALSE	cg00342530	Hypomethylated
C17orf73	17	46200721	FALSE	cg18490846	Hypomethylated
SMPDL3B	1	28134139	FALSE	cg00605270	Hypomethylated
MOCS1	6	40003649	FALSE	cg03422350	Hypomethylated
PLA2G1B	12	119250101	FALSE	cg18133966	Hypomethylated
FGF7	15	47503539	FALSE	cg27525902	Hypomethylated
LTC4S	5	179153696	FALSE	cg11394785	Hypomethylated
MBD3L1	19	8814473	FALSE	cg13727946	Hypomethylated
C1orf38	1	28078513	FALSE	cg08623383	Hypomethylated
PKD1	16	2126256	TRUE	cg18130100	Hypomethylated
C10orf10	10	44794323	FALSE	cg17186163	Hypomethylated
C7	5	40945289	FALSE	cg09179845	Hypomethylated
CXCL10	4	77163809	FALSE	cg01288089	Hypomethylated
PGAM2	7	44071959	FALSE	cg23616741	Hypomethylated
SNAPC4	9	138413179	FALSE	cg24812167	Hypomethylated
SLCO2B1	11	74539775	FALSE	cg27104271	Hypomethylated
HLA-DPA1	6	33148892	FALSE	cg13906813	Hypomethylated
YPEL4	11	57174550	FALSE	cg22705929	Hypomethylated
FLJ23447	19	13910018	FALSE	cg07425555	Hypomethylated
PRAP1	10	135010898	FALSE	cg03743584	Hypomethylated
LAG3	12	6752344	FALSE	cg01820374	Hypomethylated

GUP1	3	42718798	FALSE	cg00407150	Hypomethylated
PIB5PA	22	29848860	FALSE	cg27324619	Hypomethylated
TMC4	19	59368560	FALSE	cg01427567	Hypomethylated
CNFN	19	47586293	TRUE	cg12973651	Hypomethylated
FPRL2	19	56989780	FALSE	cg06784466	Hypomethylated
SP2	17	43347121	FALSE	cg14360917	Hypomethylated
INPP5B	1	38185275	FALSE	cg10784030	Hypomethylated
TRIM38	6	26070546	FALSE	cg22502502	Hypomethylated
TSP50	3	46734453	TRUE	cg19668234	Hypomethylated
CREB5	7	28305282	FALSE	cg10822172	Hypomethylated
CCR1	3	46224989	FALSE	cg12441928	Hypomethylated
PAX1	20	21627843	FALSE	cg24401441	Hypomethylated
ID3	1	23758996	TRUE	cg19532954	Hypomethylated
OASL	12	119961103	FALSE	cg24997562	Hypomethylated
SYNPO	5	149999796	FALSE	cg12172720	Hypomethylated
LUM	12	90029328	FALSE	cg10401088	Hypomethylated
MMP19	12	54522914	FALSE	cg16725130	Hypomethylated
KCNN4	19	48977137	FALSE	cg15977816	Hypomethylated
ABCA6	17	64649548	FALSE	cg22081096	Hypomethylated
TRIM38	6	26071421	FALSE	cg21844956	Hypomethylated
CARD6	5	40877336	FALSE	cg16250754	Hypomethylated
LRRC17	7	102340529	FALSE	cg02724472	Hypomethylated
TNFRSF18	1	1132078	TRUE	cg07671976	Hypomethylated
BPI	20	36364926	FALSE	cg15494458	Hypomethylated
IRGC	19	48912054	FALSE	cg26251865	Hypomethylated
NURIT	13	45173353	FALSE	cg16140179	Hypomethylated
NR1H4	12	99391640	FALSE	cg15381313	Hypomethylated
BTN3A3	6	26548763	FALSE	cg25193278	Hypomethylated
SH3BGR	21	39739515	TRUE	cg19946699	Hypomethylated
MYO1F	19	8548301	FALSE	cg27582235	Hypomethylated
TFEC	7	115458040	FALSE	cg04663487	Hypomethylated
PTPRH	19	60413450	FALSE	cg07097098	Hypomethylated
IL32	16	3055553	FALSE	cg18350391	Hypomethylated
TNFRSF1A	12	6321391	FALSE	cg00973286	Hypomethylated
GSTA3	6	52881670	FALSE	cg02075593	Hypomethylated
ADAMTSL5	19	1463314	FALSE	cg04601137	Hypomethylated
SLC15A2	3	123095646	TRUE	cg10523671	Hypomethylated
RPE65	1	68688486	FALSE	cg11724759	Hypomethylated
FBXW10	17	18588052	FALSE	cg10762615	Hypomethylated
CYP4F12	19	15644791	FALSE	cg05722906	Hypomethylated
G1P3	1	27871290	FALSE	cg20227766	Hypomethylated
SLC22A14	3	38322396	FALSE	cg16558203	Hypomethylated
TNFSF13B	13	107719053	FALSE	cg09646392	Hypomethylated
FLJ20581	16	20328217	FALSE	cg10938486	Hypomethylated
IBSP	4	88939570	FALSE	cg03296929	Hypomethylated
FAM12B	14	20306146	FALSE	cg12376406	Hypomethylated
ATN1	12	6904172	FALSE	cg01437411	Hypomethylated

MRVI1	11	10672171	FALSE	cg24365867	Hypomethylated
ORM1	9	116124423	FALSE	cg12927617	Hypomethylated
AMBN	4	71492427	FALSE	cg13523386	Hypomethylated
P2RX7	12	120055258	TRUE	cg07602200	Hypomethylated
NR5A1	9	126309628	TRUE	cg00691625	Hypomethylated
SMPDL3B	1	28133954	FALSE	cg22421699	Hypomethylated
XLKD1	11	10546858	FALSE	cg03908676	Hypomethylated
CD209	19	7718265	FALSE	cg01618851	Hypomethylated
BRD4	19	15252124	FALSE	cg02533173	Hypomethylated
PLA2G4E	15	40077231	FALSE	cg15228639	Hypomethylated
TPM3	1	152431575	FALSE	cg12205230	Hypomethylated
KCNQ1	11	2784940	TRUE	cg16609872	Hypomethylated
LONRF2	2	100293082	FALSE	cg12232463	Hypomethylated
PHKG1	7	56128022	FALSE	cg05724065	Hypomethylated
CASP10	2	201756345	FALSE	cg12105450	Hypomethylated
DSPG3	12	89924248	FALSE	cg13798376	Hypomethylated
FHL2	2	105421438	FALSE	cg10635061	Hypomethylated
EBI3	19	4180509	FALSE	cg19529363	Hypomethylated
KIAA0020	9	2828508	FALSE	cg24471894	Hypomethylated
ANGPTL2	9	128925551	FALSE	cg09427311	Hypomethylated
FLJ42393	3	189378916	FALSE	cg21909391	Hypomethylated
SLC15A2	3	123096393	FALSE	cg18636558	Hypomethylated
SFTPD	10	81698971	FALSE	cg03600318	Hypomethylated
LYST	1	234113657	FALSE	cg12167564	Hypomethylated
INHBC	12	56114782	FALSE	cg08640498	Hypomethylated
ACOT11	1	54786297	FALSE	cg10266490	Hypomethylated
PIB5PA	22	29848963	FALSE	cg18053607	Hypomethylated
CMA1	14	24047613	FALSE	cg08020808	Hypomethylated
GIMAP1	7	150044613	FALSE	cg12914657	Hypomethylated
TAGAP	6	159386446	FALSE	cg05103623	Hypomethylated
C1orf85	1	154531899	FALSE	cg22377142	Hypomethylated
C10orf26	10	104526111	FALSE	cg09405083	Hypomethylated
LGP2	17	37518341	FALSE	cg16762195	Hypomethylated
JPH4	14	23118395	FALSE	cg06536578	Hypomethylated
G6PC	17	38306255	FALSE	cg26673195	Hypomethylated
IL7R	5	35892887	FALSE	cg04312209	Hypomethylated
OR2W1	6	29121315	FALSE	cg25133685	Hypomethylated
DARC	1	157441234	FALSE	cg18552413	Hypomethylated
FLJ25422	5	36337851	FALSE	cg24237439	Hypomethylated
PTPN7	1	200397543	FALSE	cg23506842	Hypomethylated
GBGT1	9	135028511	TRUE	cg01169778	Hypomethylated
FLJ34503	6	114331999	FALSE	cg19815376	Hypomethylated
LHX3	9	138237697	FALSE	cg14091657	Hypomethylated
RARRES3	11	63060913	FALSE	cg25599242	Hypomethylated
CFHR4	1	195010295	FALSE	cg04614339	Hypomethylated
FAM107A	3	58538309	FALSE	cg06638451	Hypomethylated
CST9L	20	23497455	FALSE	cg27655855	Hypomethylated

PLAT	8	42183830	FALSE	cg22038738	Hypomethylated
GBP1	1	89303252	FALSE	cg13406950	Hypomethylated
KCNG4	16	82830658	FALSE	cg11913615	Hypomethylated
ATN1	12	6903824	FALSE	cg22475430	Hypomethylated
IFI35	17	38412815	FALSE	cg08090640	Hypomethylated
GSTA3	6	52883168	FALSE	cg04340502	Hypomethylated
RHD	1	25471465	FALSE	cg06388544	Hypomethylated
GIMAP2	7	150013657	FALSE	cg25918245	Hypomethylated
SLC18A1	8	20084934	FALSE	cg22441882	Hypomethylated
TRIP6	7	100303561	FALSE	cg01274660	Hypomethylated
PTH	11	13475601	FALSE	cg24816298	Hypomethylated
LYST	1	234113562	FALSE	cg02829654	Hypomethylated
SLC12A6	15	32398301	FALSE	cg14307212	Hypomethylated
OLFML3	1	114323095	FALSE	cg20618842	Hypomethylated
GPR44	11	60379720	FALSE	cg04754011	Hypomethylated
NRIP2	12	2814741	FALSE	cg05194726	Hypomethylated
MYOZ3	5	150020473	FALSE	cg14600885	Hypomethylated
CYP2A7	19	46081197	FALSE	cg25427638	Hypomethylated
KLK10	19	56216502	FALSE	cg19356189	Hypomethylated
MRGPRX2	11	19038166	FALSE	cg22051636	Hypomethylated
KLF11	2	10101754	TRUE	cg20389709	Hypomethylated
P518	9	132758787	TRUE	cg03924115	Hypomethylated
NAT8	2	73723682	FALSE	cg09172980	Hypomethylated
ID2	2	8739739	TRUE	cg13055278	Hypomethylated
GGTLA1	22	22971654	FALSE	cg15448245	Hypomethylated
ADORA1	1	201325577	FALSE	cg11719784	Hypomethylated
DEFA1	8	6825434	FALSE	cg17267907	Hypomethylated
TAT	16	70168544	FALSE	cg22136365	Hypomethylated
CLEC4C	12	7794437	FALSE	cg01120761	Hypomethylated
FLJ45964	2	240165186	FALSE	cg17977362	Hypomethylated
