# Supplementary material for: Epigenetic Disruption of the PIWI Pathway in Human Spermatogenic Disorders
Source: PLoS One. 2012 Oct 24;7(10):e47892. doi: 10.1371/journal.pone.0047892 (PMC3480440; doi:10.1371/journal.pone.0047892)
Supplement: Table S3 — Gene ontology analysis of differentially methylated genes in SpF. (PDF) [file pone.0047892.s005.pdf]

Supplementary table S3: Gene ontology analysis of differentially methylated genes in SpF.

Hypermethylated in SpF

Term	Fisher Exact	GO category
female pregnancy	8,2E-5	Biological process (3)
germ-line stem cell maintenance	1,6E-4	Biological process (3)
digestive system process	9,2E-3	Biological process (3)
ovulation cycle	1,1E-2	Biological process (3)
sex differentiation	1,3E-2	Biological process (3)
male sex differentiation	1,4E-2	Biological process (3)
ovarian follicle development	1,5E-2	Biological process (3)
regulation of cell size	1,7E-2	Biological process (3)
reproductive cellular process	1,8E-2	Biological process (3)
reproductive process in a multicellular organism	2,2E-2	Biological process (3)
gamete generation	3,0E-2	Biological process (3)
mitochondrion organization	3,2E-2	Biological process (3)
circulatory system process	3,2E-2	Biological process (3)
secretion	3,9E-2	Biological process (3)
negative regulation of cell proliferation	4,2E-2	Biological process (3)
male gamete generation	4,4E-2	Biological process (3)

Term	Fisher Exact	GO category
germ-line stem cell maintenance	1,5E-4	Biological process (5)
pancreatic juice secretion	9,0E-4	Biological process (5)
positive regulation of myeloid leukocyte differentiation	1,3E-3	Biological process (5)
negative regulation of mitotic cell cycle	2,4E-3	Biological process (5)
stem cell maintenance	3,5E-3	Biological process (5)
stem cell development	3,9E-3	Biological process (5)
gene silencing	5,0E-3	Biological process (5)
positive regulation of myeloid cell differentiation	7,7E-3	Biological process (5)
male sex differentiation	1,3E-2	Biological process (5)
cartilage development	1,3E-2	Biological process (5)
ovarian follicle development	1,4E-2	Biological process (5)
regulation of myeloid leukocyte differentiation	1,4E-2	Biological process (5)
meiosis I	1,5E-2	Biological process (5)
regulation of gene expression, epigenetic	1,5E-2	Biological process (5)
ribonucleotide biosynthetic process	1,9E-2	Biological process (5)
nucleobase, nucleoside, nucleotide and nucleic acid biosynthetic process	3,3E-2	Biological process (5)
nucleobase, nucleoside and nucleotide	3,3E-2	Biological process (5)

biosynthetic process		
negative regulation of cell proliferation	3,5E-2	Biological process (5)
spermatogenesis	3,8E-2	Biological process (5)

Hypomethylated in SpF

Term	Fisher Exact	GO category
defense response	1,2E-7	Biological process (3)
regulation of immune system process	2,8E-7	Biological process (3)
taxis	7,5E-7	Biological process (3)
chemotaxis	7,5E-7	Biological process (3)
positive regulation of biological process	7,8E-7	Biological process (3)
positive regulation of cellular process	1,1E-6	Biological process (3)
cell chemotaxis	1,5E-6	Biological process (3)
locomotory behavior	1,5E-5	Biological process (3)
leukocyte chemotaxis	1,6E-5	Biological process (3)
regulation of response to stimulus	2,0E-5	Biological process (3)
regulation of multicellular organismal process	2,1E-5	Biological process (3)
regulation of immune response	3,3E-5	Biological process (3)
regulation of leukocyte activation	3,7E-5	Biological process (3)
response to wounding	4,1E-5	Biological process (3)
positive regulation of response to stimulus	5,2E-5	Biological process (3)
positive regulation of immune system process	5,6E-5	Biological process (3)
positive regulation of protein transport	5,9E-5	Biological process (3)
positive regulation of immune response	6,1E-5	Biological process (3)
regulation of cell activation	6,1E-5	Biological process (3)
regulation of cellular localization	8,6E-5	Biological process (3)
immune effector process	1,7E-4	Biological process (3)
negative regulation of immune system process	2,3E-4	Biological process (3)
regulation of transport	2,7E-4	Biological process (3)
homeostatic process	3,2E-4	Biological process (3)
positive regulation of protein secretion	3,3E-4	Biological process (3)
regulation of cytokine production	3,7E-4	Biological process (3)
positive regulation of cell proliferation	4,6E-4	Biological process (3)
positive regulation of immune effector process	4,7E-4	Biological process (3)
regulation of leukocyte mediated cytotoxicity	4,7E-4	Biological process (3)
positive regulation of transport	5,1E-4	Biological process (3)
multicellular organismal catabolic process	5,5E-4	Biological process (3)
regulation of cell killing	7,3E-4	Biological process (3)
regulation of localization	7,6E-4	Biological process (3)
cell volume homeostasis	1,0E-3	Biological process (3)
regulation of protein localization	1,1E-3	Biological process (3)
humoral immune response	1,1E-3	Biological process (3)
phospholipid translocation	1,3E-3	Biological process (3)
response to other organism	1,3E-3	Biological process (3)

regulation of cell proliferation	1,5E-3	Biological process (3)
negative regulation of leukocyte activation	1,5E-3	Biological process (3)
leukocyte mediated immunity	1,7E-3	Biological process (3)
negative regulation of cell activation	2,1E-3	Biological process (3)
response to bacterium	2,4E-3	Biological process (3)
tissue homeostasis	2,4E-3	Biological process (3)
regulation of response to external stimulus	2,6E-3	Biological process (3)
regulation of response to stress	2,7E-3	Biological process (3)
positive regulation of homeostatic process	2,8E-3	Biological process (3)
positive regulation of calcium ion transport into cytosol	3,1E-3	Biological process (3)
negative regulation of glutamate secretion	3,2E-3	Biological process (3)
positive regulation of ion transport	3,4E-3	Biological process (3)
positive regulation of leukocyte mediated cytotoxicity	3,5E-3	Biological process (3)
innate immune response	4,5E-3	Biological process (3)
positive regulation of defense response	4,6E-3	Biological process (3)
cellular alkene metabolic process	4,6E-3	Biological process (3)
tissue development	4,8E-3	Biological process (3)
positive regulation of leukocyte activation	5,0E-3	Biological process (3)
positive regulation of cell killing	5,2E-3	Biological process (3)
positive regulation of secretion	5,7E-3	Biological process (3)
response to virus	5,7E-3	Biological process (3)
positive regulation of cell activation	6,2E-3	Biological process (3)
negative regulation of hormone secretion	6,5E-3	Biological process (3)
positive regulation of organelle organization	7,8E-3	Biological process (3)
regulation of binding	7,9E-3	Biological process (3)
negative regulation of lipid metabolic process	8,0E-3	Biological process (3)
lymphocyte homeostasis	8,0E-3	Biological process (3)
cell migration	8,3E-3	Biological process (3)
regulation of calcium ion transport into cytosol	8,9E-3	Biological process (3)
negative regulation of secretion	8,9E-3	Biological process (3)
leukocyte activation	1,0E-2	Biological process (3)
multicellular organismal macromolecule metabolic process	1,1E-2	Biological process (3)
negative regulation of multicellular organismal process	1,1E-2	Biological process (3)
signal transduction	1,2E-2	Biological process (3)
regulation of angiogenesis	1,4E-2	Biological process (3)
organ development	1,5E-2	Biological process (3)
positive regulation of response to external stimulus	1,5E-2	Biological process (3)
cell-substrate adhesion	1,5E-2	Biological process (3)
positive regulation of locomotion	1,5E-2	Biological process (3)
negative regulation of transport	1,5E-2	Biological process (3)
response to drug	1,5E-2	Biological process (3)
regulation of organelle organization	1,6E-2	Biological process (3)
positive regulation of macromolecule metabolic process	1,6E-2	Biological process (3)

cell motility	1,7E-2	Biological process (3)
negative regulation of biological process	1,7E-2	Biological process (3)
positive regulation of cellular component organization	1,9E-2	Biological process (3)
regulation of cell death	1,9E-2	Biological process (3)
regulation of developmental process	2,3E-2	Biological process (3)
positive regulation of cell communication	2,5E-2	Biological process (3)
cellular chemical homeostasis	2,7E-2	Biological process (3)
lymphocyte activation	2,9E-2	Biological process (3)
positive regulation of biosynthetic process	3,0E-2	Biological process (3)
positive regulation of metabolic process	3,3E-2	Biological process (3)
regulation of cell communication	3,5E-2	Biological process (3)
secretion	3,5E-2	Biological process (3)
positive regulation of cellular metabolic process	3,8E-2	Biological process (3)
transmembrane transport	4,5E-2	Biological process (3)
negative regulation of cellular process	4,9E-2	Biological process (3)

**Term**	**Fisher Exact**	**GO category**
chemotaxis	3,7E-7	Biological process (5)
cell chemotaxis	9,8E-7	Biological process (5)
leukocyte chemotaxis	1,1E-5	Biological process (5)
regulation of leukocyte activation	2,1E-5	Biological process (5)
positive regulation of immune response	3,6E-5	Biological process (5)
positive regulation of protein transport	4,0E-5	Biological process (5)
positive regulation of adaptive immune response based on somatic recombination of immune receptors built from immunoglobulin superfamily domains	5,4E-5	Biological process (5)
protein kinase cascade	6,2E-5	Biological process (5)
positive regulation of adaptive immune response	6,4E-5	Biological process (5)
positive regulation of leukocyte mediated immunity	1,0E-4	Biological process (5)
positive regulation of lymphocyte mediated immunity	1,0E-4	Biological process (5)
regulation of lymphocyte mediated immunity	1,0E-4	Biological process (5)
regulation of adaptive immune response based on somatic recombination of immune receptors built from immunoglobulin superfamily domains	1,1E-4	Biological process (5)
regulation of adaptive immune response	1,3E-4	Biological process (5)
regulation of secretion	1,3E-4	Biological process (5)
leukocyte migration	1,4E-4	Biological process (5)
negative regulation of macrophage activation	1,9E-4	Biological process (5)
regulation of protein transport	2,0E-4	Biological process (5)
regulation of leukocyte mediated immunity	2,0E-4	Biological process (5)
positive regulation of cell proliferation	2,3E-4	Biological process (5)
regulation of lymphocyte activation	2,3E-4	Biological process (5)
positive regulation of protein secretion	2,5E-4	Biological process (5)
regulation of establishment of protein localization	2,9E-4	Biological process (5)
positive regulation of transport	3,0E-4	Biological process (5)

positive regulation of immune effector process	3,5E-4	Biological process (5)
regulation of leukocyte mediated cytotoxicity	3,7E-4	Biological process (5)
regulation of immune effector process	5,3E-4	Biological process (5)
negative regulation of lipid catabolic process	6,9E-4	Biological process (5)
regulation of glutamate secretion	6,9E-4	Biological process (5)
cell volume homeostasis	8,7E-4	Biological process (5)
regulation of defense response	8,9E-4	Biological process (5)
regulation of metal ion transport	9,4E-4	Biological process (5)
defense response to bacterium	9,8E-4	Biological process (5)
lipid translocation	1,1E-3	Biological process (5)
phospholipid translocation	1,1E-3	Biological process (5)
negative regulation of interleukin-2 biosynthetic process	1,1E-3	Biological process (5)
negative regulation of leukocyte activation	1,2E-3	Biological process (5)
regulation of T cell activation	1,3E-3	Biological process (5)
regulation of protein secretion	1,3E-3	Biological process (5)
negative regulation of cell activation	1,6E-3	Biological process (5)
tissue homeostasis	1,8E-3	Biological process (5)
regulation of interleukin-2 biosynthetic process	2,2E-3	Biological process (5)
positive regulation of homeostatic process	2,3E-3	Biological process (5)
positive regulation of lymphocyte activation	2,4E-3	Biological process (5)
tissue development	2,4E-3	Biological process (5)
acute inflammatory response	2,5E-3	Biological process (5)
positive regulation of calcium ion transport into cytosol	2,6E-3	Biological process (5)
regulation of ion transport	2,6E-3	Biological process (5)
positive regulation of ion transport	2,7E-3	Biological process (5)
peptide cross-linking via chondroitin 4-sulfate glycosaminoglycan	2,8E-3	Biological process (5)
negative regulation of glutamate secretion	2,8E-3	Biological process (5)
cytokine-mediated signaling pathway	2,9E-3	Biological process (5)
positive regulation of leukocyte mediated cytotoxicity	3,0E-3	Biological process (5)
regulation of transcription factor activity	3,2E-3	Biological process (5)
positive regulation of defense response	3,5E-3	Biological process (5)
positive regulation of leukocyte activation	3,7E-3	Biological process (5)
response to mercury ion	3,9E-3	Biological process (5)
positive regulation of T cell activation	4,2E-3	Biological process (5)
regulation of inflammatory response	4,2E-3	Biological process (5)
positive regulation of secretion	4,2E-3	Biological process (5)
positive regulation of cell activation	4,6E-3	Biological process (5)
cell migration	5,5E-3	Biological process (5)
negative regulation of hormone secretion	5,5E-3	Biological process (5)
regulation of lipid catabolic process	5,5E-3	Biological process (5)
unsaturated fatty acid metabolic process	5,5E-3	Biological process (5)
regulation of lymphocyte proliferation	6,1E-3	Biological process (5)
positive regulation of organelle organization	6,1E-3	Biological process (5)
regulation of leukocyte proliferation	6,4E-3	Biological process (5)

regulation of mononuclear cell proliferation	6,4E-3	Biological process (5)
positive regulation of transferase activity	6,7E-3	Biological process (5)
negative regulation of lipid metabolic process	6,8E-3	Biological process (5)
positive regulation of lymphocyte proliferation	7,3E-3	Biological process (5)
negative regulation of secretion	7,3E-3	Biological process (5)
regulation of calcium ion transport into cytosol	7,5E-3	Biological process (5)
complement activation, classical pathway	7,5E-3	Biological process (5)
blood vessel development	7,6E-3	Biological process (5)
positive regulation of leukocyte proliferation	7,7E-3	Biological process (5)
positive regulation of mononuclear cell proliferation	7,7E-3	Biological process (5)
hexose transport	8,3E-3	Biological process (5)
regulation of interleukin-2 production	8,3E-3	Biological process (5)
positive regulation of inflammatory response	8,3E-3	Biological process (5)
regulation of cytokine secretion	8,3E-3	Biological process (5)
positive regulation of macromolecule metabolic process	8,5E-3	Biological process (5)
positive regulation of signal transduction	8,6E-3	Biological process (5)
regulation of apoptosis	8,7E-3	Biological process (5)
vasculature development	8,8E-3	Biological process (5)
positive regulation of protein kinase cascade	9,1E-3	Biological process (5)
monosaccharide transport	9,1E-3	Biological process (5)
regulation of programmed cell death	9,6E-3	Biological process (5)
blood vessel morphogenesis	9,7E-3	Biological process (5)
positive regulation of macromolecule biosynthetic process	1,0E-2	Biological process (5)
positive regulation of calcium ion transport	1,1E-2	Biological process (5)
regulation of amine transport	1,1E-2	Biological process (5)
regulation of angiogenesis	1,2E-2	Biological process (5)
negative regulation of transport	1,2E-2	Biological process (5)
regulation of cytoskeleton organization	1,2E-2	Biological process (5)
positive regulation of response to external stimulus	1,2E-2	Biological process (5)
positive regulation of phosphorus metabolic process	1,3E-2	Biological process (5)
positive regulation of phosphate metabolic process	1,3E-2	Biological process (5)
regulation of interleukin-6 production	1,4E-2	Biological process (5)
leukocyte homeostasis	1,4E-2	Biological process (5)
negative regulation of catabolic process	1,5E-2	Biological process (5)
positive regulation of cellular biosynthetic process	1,6E-2	Biological process (5)
lymphocyte mediated immunity	1,7E-2	Biological process (5)
positive regulation of DNA binding	1,7E-2	Biological process (5)
positive regulation of cell communication	1,7E-2	Biological process (5)
positive regulation of biosynthetic process	1,8E-2	Biological process (5)
cellular chemical homeostasis	1,8E-2	Biological process (5)
regulation of cytokine biosynthetic process	2,0E-2	Biological process (5)
positive regulation of cellular metabolic process	2,1E-2	Biological process (5)
response to lipopolysaccharide	2,3E-2	Biological process (5)
ion homeostasis	2,9E-2	Biological process (5)
T cell activation	3,2E-2	Biological process (5)

positive regulation of gene expression	3,3E-2	Biological process (5)
endocytosis	3,5E-2	Biological process (5)
positive regulation of transcription, DNA-dependent	3,6E-2	Biological process (5)
positive regulation of nitrogen compound metabolic process	3,6E-2	Biological process (5)
positive regulation of RNA metabolic process	3,8E-2	Biological process (5)
regulation of signal transduction	3,8E-2	Biological process (5)
epithelium development	4,0E-2	Biological process (5)
