# Supplementary material for: Epigenetic Disruption of the PIWI Pathway in Human Spermatogenic Disorders
Source: PLoS One. 2012 Oct 24;7(10):e47892. doi: 10.1371/journal.pone.0047892 (PMC3480440; doi:10.1371/journal.pone.0047892)
Supplement: Table S4 — Differentially methylated gene promoters overlapping piRNAs. (PDF) [file pone.0047892.s006.pdf]

Supplementary table S4: Differentially methylated gene promoters overlapping piRNAs.

Gene symbol	piRNA	piRNA start	piRNA end	Transcription start site
DPM1	DQ600332	49575937	49575966	49575060
GPR156	DQ601878	119965297	119965325	119963325
HIST1H2AA	DQ583568	25727116	25727142	25726291
HIST1H2AA	DQ583569	25727116	25727143	25726291
HIST1H2AA	DQ583570	25727116	25727144	25726291
HIST1H2AA	DQ577170	25727151	25727181	25726291
HIST1H2AA	DQ571051	25727508	25727538	25726291
IL16	DQ578799	81588605	81588631	81589268
IL16	DQ592408	81590178	81590207	81591720
KLK1	DQ575553	51328726	51328755	51327043
RAB24	DQ576332	176731696	176731722	176728199
RAB24	DQ576332	176731696	176731722	176728199
SMPD3	DQ594877	68483599	68483628	68482409
